# Spark: sparse hierarchical energy minimization for scalable prediction of RNA pseudoknots

**DOI:** 10.1093/bioinformatics/btag194

**Published:** 2026-04-21

**Authors:** Mateo Gray, Sebastian Will, Hosna Jabbari

**Affiliations:** Department of Biomedical Engineering, University of Alberta, Edmonton, Alberta, T6G 2R3, Canada; Laboratoire d’informatique de l’École polytechnique (LIX), Institut Polytechnique de Paris, 91120 Palaiseau, France; Department of Biomedical Engineering, University of Alberta, Edmonton, Alberta, T6G 2R3, Canada

## Abstract

**Motivation:**

The biological functions of RNAs are tightly connected to their specific RNA structures. As experimental techniques to determine high-accuracy structures are costly and time-consuming, computational prediction approaches became indispensable for biological RNA research; most notably, the prediction of minimum free energy secondary structures. Pseudoknots are prevalent, highly significant structural motifs, yet they are commonly ignored to achieve acceptable efficiency. Existing reliable pseudoknot prediction methods typically have prohibitive complexity. A route to fast scalable pseudoknot prediction was suggested with HFold following the hierarchical folding hypothesis. Recent successful sparsification of the CCJ pseudoknot prediction algorithm in Knotty promises a further boost by introducing this technique to hierarchical folding.

**Results:**

We introduce Spark, a sparsified algorithm for predicting pseudoknotted RNA structures. Spark predicts exactly the same minimum-energy structures as its predecessor HFold in the accurate HotKnots 2.0 energy model for pseudoknots. While sparsification maintains exact energy minimization and theoretical complexity, it strongly improves the time and space consumption over HFold. We benchmarked the performance of Spark against HFold and, as a pseudoknot-free baseline, RNAfold. Compared with HFold, Spark substantially reduces both run time and memory usage, while achieving run times close to RNAfold. Across all tested sequence lengths, Spark used the least memory and consistently ran faster than HFold.

**Conclusion:**

Combining sparsification and hierarchical folding in Spark results in an remarkably fast and memory-efficient tool for the accurate prediction of pseudoknotted RNA structures. Consequently, Spark practically enables pseudoknot prediction in large scale and even for very long RNA sequences.

**Availability:**

Spark software is available on Github (https://github.com/TheCOBRALab/Spark), with a permanent archive of the software and results deposited on Zenodo (https://doi.org/10.5281/zenodo.19073315).

## 1 Introduction

RNA molecules carry out essential biological functions that often depend critically on their specific structural conformations (Kozak 2005, Cruz and Westhof 2009, Warf and Berglund 2010, Mortimer et al. 2014, Wilson and Lilley 2015). Accordingly, a thorough understanding of many biological systems demands accurate models of RNA structure. Because experimental determination of RNA conformations is both costly and time-consuming, computational methods have become indispensable. These approaches not only complement experimental techniques by generating rapid structural hypotheses, but also enable new lines of inquiry, such as probing thermodynamic ensembles, exploring folding kinetics, and pursuing the rational design of functional RNAs.

A promising class of computational methods focuses on RNA secondary structure, where structure prediction can be formalized as combinatorial optimization problem of finding minimum free energy (MFE) structures. These approaches profit from energy models with accurate empirically determined energy parameters (Matthews et al. 2004), known as nearest neighbor models or more specifically, the “Turner model,” leveraged by efficient dynamic programming optimization algorithms. Consequently, they can predict realistic RNA secondary structures with high accuracy.

However, most practically applied algorithms in this class are restricted to pseudoknot-free structures, even though pseudoknots are common in RNAs. This limitation reduces their accuracy and constrains their range of applications.

### 1.1 Exact and accurate pseudoknot prediction

To predict pseudoknotted RNA secondary structures with high accuracy, several advanced dynamic-programming algorithms have been developed (Rivas and Eddy 1999, Jabbari et al. 2018).

These methods optimize energy in pseudoknot models such as HotKnots 2.0 (Dirks and Pierce 2003, Ren et al. 2005). HotKnots 2.0 extends the well-established Turner nearest-neighbor model by incorporating empirically trained pseudoknot parameters. Nevertheless, these methods are often limited by extreme computational complexity, for example $O(n^{6})$ time and $O(n^{4})$ space in the sequence length *n* (Rivas and Eddy 1999), and in some cases still support only simple pseudoknots, for example (Dirks and Pierce 2003), which runs in $O(n^{5})$ time.

While heuristics, such as the HotKnots method (Ren et al. 2005), have been developed to overcome these computational limitations, we advocate the use of exact combinatorial methods that provide controlled and precisely defined solutions. By restricting attention to carefully chosen classes of pseudoknots and applying targeted algorithmic optimizations–most notably sparsification–we can achieve both computational tractability and broad practical utility.

We previously demonstrated this principle with the CCJ algorithm (Chen et al. 2009) and its sparsified successor Knotty. Space-efficient sparsification of the CCJ recurrences enables Knotty to compute MFE structures containing complex motifs, such as 4-chain pseudoknots and kissing hairpins with arbitrarily nested substructures of the same complexity (Jabbari et al. 2018).

**Figure 1:**
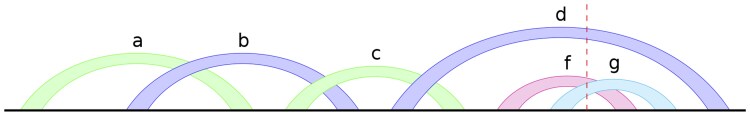
Band configuration of a density-2 structure. Bands and density-2 are elaborated in Jabbari et al. (2017, 2018); here, we recall their definitions. The pseudoknotted base pairs of a structure can be partitioned into bands; all base pairs within one band are mutually nested and cross the exact same other base pairs of the structure. A set of bands can be further decomposed into components of transitively crossing bands, here {a, b, c, d} and {f, g}. The density-*k* property requires that no position is covered by more than *k* base pairs of the same component. For example, the position at the dashed red line satisfies density-2, since it is covered by the bands *d*, *f* and *g*, where only two of them are from the same component.

To further improve efficiency, we introduced hierarchical pseudoknot prediction in HFold (Jabbari et al. 2007, 2008). HFold is fast enough to serve as the backbone of meta-strategies (Jabbari and Condon 2014) and to fold long RNA sequences. It predicts a broad, biologically relevant class of potentially pseudoknotted structures, called the *density-2 class*, a special class of bi-secondary structures (Witwer et al. 2004); see Figure 1 and Jabbari et al. (2017, 2018). Given a pseudoknot-free (pk-free) secondary structure *G*, HFold predicts a second pk-free structure $G^{\prime }$, compatible with the first structure, such that the combined *density-2* structure is energetically optimal given *G*.

### 1.2 Contributions

Further extending pseudoknot prediction capabilities, we introduce the sparsified dynamic programming algorithm Spark to predict hierarchically constrained pseudoknots. Spark solves exactly the same problem as HFold, namely, the hierarchical prediction of pseudoknotted structures using a realistic pseudoknot energy model (HotKnots 2.0). While Spark preserves the asymptotic complexity of HFold, it substantially improves practical run time and space consumption through sparsification.

We outline the recurrences of the novel algorithm Spark, contrasting them with those of HFold and focusing on the requirements of sparsification. We prove the correctness of the sparsification and illustrate the algorithm using graphical notation (see Fig. 3). Finally, we empirically demonstrate the improvements over HFold and compare the results to RNAfold for reference.

### 1.3 Further remarks on sparsification

Sparsification was originally proposed as a non-heuristic approach to improve run time (Wexler et al. 2007) and even space efficiency (Backofen et al. 2011) for pk-free RNA secondary structure prediction. It has since been applied to more complex prediction tasks, such as interaction prediction by Salari et al. (2010). As we showed previously, sparsification can reduce run time and space complexity even when using realistic RNA energy models (Will and Jabbari 2016) and when accounting for dangling ends, as in SparseRNAFolD by Gray et al. (2024). In the latter work, we closed a significant gap in the use of sparsification with realistic energy models by demonstrating sparsified MFE folding with dangling ends for the pk-free case. This allowed us to surpass RNAfold (Lorenz et al. 2011) for the equivalent task.

As its key idea, sparsification limits the minimization cases in the dynamic programming recursions to only *candidate* subproblems. This is achieved by omitting non-candidate cases that *provably* are not required to compute the exact minima. In predicting the (pk-free) MFE structure given a sequence *S*, sparsification exploits the subadditivity of the MFE $W_{i,j}$ of subsequences from *i* to *j*; more specifically, it uses the inverse triangle inequality $W_{i,j}\leq W_{i,k}+W_{k+1,j}$ (Wexler et al. 2007) implied by the additive energy model.

For pk-free MFE prediction, sparsification can thus reduce the time complexity from $O(n^{3})$ to $O(n^{2}+nZ)$, where *Z* is the number of candidates (Wexler et al. 2007). Will and Jabbari (2016) showed that, in a realistic energy model, energy minimization only requires storing the candidates and a linear number of subproblems. In the nearest-neighbor energy model, the optimal structure can then be reconstructed by recording a set of trace arrows, typically of small size *T*, leading to a space complexity of O(n+Z+T). This result was further extended to include energy contributions from dangling ends in Gray et al. (2024). Our novel algorithm Spark achieves the same time complexity and a similar space complexity for pseudoknot MFE prediction.

## 2 Preliminaries and review of the hierarchical pseudoknot prediction in HFold

We will first review HFold’s non-sparse dynamic programming algorithm (Jabbari et al. 2007, 2008). This prepares our later description of the sparsified Spark algorithm, which requires the same preliminaries and follows the same overall decomposition scheme.

We define an *RNA sequence* of length *n* as $S=s_{1}s_{2}\ldots s_{n}$, where each $s_{k}$, 1≤k≤n, is a nucleotide in A,C,G,U. We refer to each nucleotide (base) by its index. A *secondary structure* is a set of base pairs (i,j), 1≤i<j≤n, such that each index occurs in at most one base pair (i.e. no triplets). A structure that contains *crossing* base pairs (i,j) and $\left(i^{\prime },j^{\prime }\right)$, where $i<i^{\prime }<j<j^{\prime }$ or $i^{\prime }<i<j^{\prime }<j$, is called *pseudoknotted*; otherwiase, it is *pseudoknot-free* (*pk-free*) or *non-crossing*.

Given an RNA sequence *S* and a pk-free secondary structure *G*, HFold computes a second, disjoint pk-free structure $G^{\prime }$ such that the combined structure $G\cup G^{\prime }$ is density-2 and its energy is minimized, conditional on *G*.

In a density-2 structure, the terminal positions *i* and *j* of any substructure can close a loop in exactly two ways. (1) The nucleotides at positions *i* and *j* form a single base pair (i,j), thereby completing an ordinary, non-crossing loop. (2) The endpoints are connected by a *chain* of crossing base pairs $i_{1}.j_{1},i_{2}.j_{2},\ldots ,i_{k}.j_{k}\;(k>1)$ ordered so that $i_{1}<i_{2}<\ldots <i_{k}<j_{1}<j_{2}<\ldots <j_{k}$ with $i=i_{1}$ and $j=j_{k}$. This sequence of crossings closes a *pseudoloop*. Substructures that are neither *loop-closed* nor *pseudoloop-closed* can be split into two disjoint substructures.

**Figure 2:**
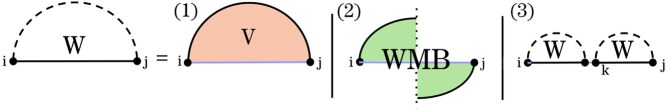
HFold’s $W_{i,j}$ recurrence in graphical notation. (i) (i,j) closes a regular loop, $V_{i,j}$; (ii) *i* and *j* are connected through a chain of crossing base pairs and close a pseudoloop, $WMB_{i,j}$; and (iii) the structure over the region [i,j] can be decomposed into two disjoint substructures.

**Figure 3:**
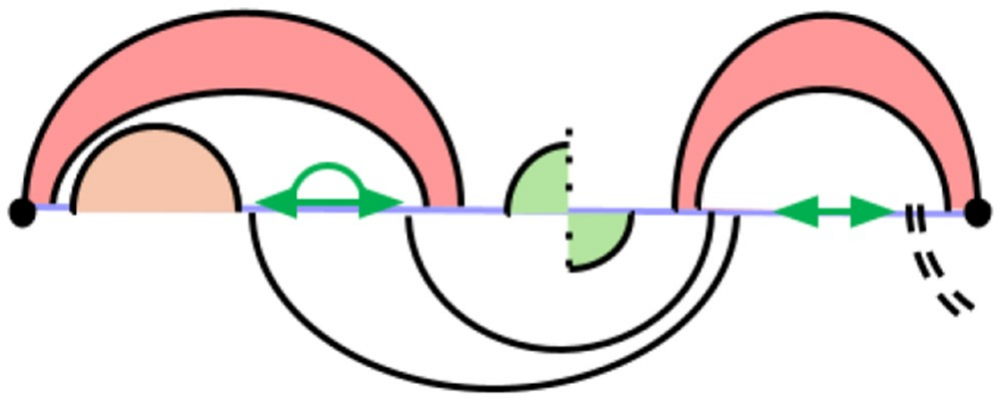
Graphical notation reference. This notation is used consistently in all illustrations of the recurrences of Spark and HFold. Dashed arcs indicate possible structure, and each solid arc represents a base pair. The vertical dotted line denotes an overlapping chain of bands of arbitrary length and indicates that the chain can begin or end in *G* (above the horizontal line) or $G^{\prime }$ (below the horizontal line). Filled arcs show regions covered by specific structure classes, for example orange for *V* and green for *WMB*. Green bidirectional arrows represent a nested structure inside a pseudoknot (*WI*), whereas arrows that include an arc represent a nested structure inside a band ($WI^{\prime }$).

Figure 2 illustrates this decomposition of general subproblems. Here and in the following, we systematically use the graphical notation introduced in Fig. 3. The decomposition in Fig. 2 allows us to compute $W_{i,j}$, the minimum free energy of structures on the subsequence $S_{i..j}=s_{i}s_{i+1}\ldots s_{j}$, by minimizing over all possible cases. The HFold algorithm delegates the first two cases, where the substructure is, respectively, loop-closed or pseudoloop-closed, to additional recurrences $V_{i,j}$ and $WMB_{i,j}$. Here, $V_{i,j}$ denotes the MFE of structures on the subsequence $S_{i..j}$ that contain the base pair (i,j), and $WMB_{i,j}$ denotes the MFE of structures that contain the chain of base pairs connecting *i* to *j*. In the remaining cases, the optimal structure can be decomposed into general MFE structures on a prefix $S_{i..k}$ and a suffix $S_{k+1..j}$ for some i<k<j.

**Figure 4:**
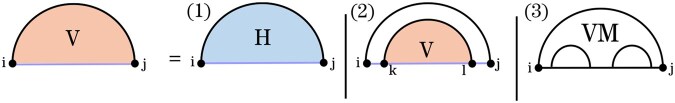
Recurrence $V_{i,j}$ for loop-closed structures. In Case 1, (i,j) closes a hairpin loop, H  ${\;}_{i,j}$; in Case 2, base pairs (i,j) and (k,l) close an internal loop, I  ${\;}_{i,k,l,j}$; and in Case 3, (i,j) closes a multiloop, $VM_{i,j}$.

For a single non-crossing base pair (i,j), let $V_{i,j}$ denote the MFE of all substructures closed by (i,j). This value is obtained as the minimum over three cases: (i,j) closes (1) a hairpin loop, (2) an interior loop with inner base pair (k,l), or (3) a multiloop (Fig. 4).

For the MFE contribution of a multiloop, HFold introduces an additional recurrence, $VM_{i,j}$. We do not repeat the details from the original publications, as this case closely resembles the pseudoknot-free multiloop formulation. We note, however, that it supports recursive pseudoknots by recursing to our $V_{i,j}$ and $WMB_{i,j}$.

**Figure 5:**
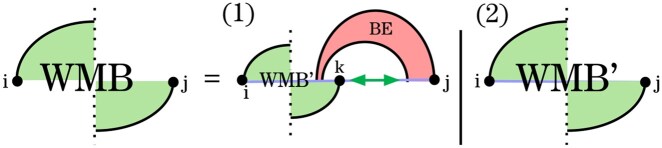
HFold recurrence $WMB_{i,j}$ for closed pseudoloops. In Case 1, there is a base pair $\left(j^{\prime },j\right)$ in *G* that defines a band. Removing it yields a pseudoloop prefix $WMB_{i,k}$ for some *k* and a recursive substructure (scored by *WI*, green arrow). All remaining substructures are delegated to $WMB^{\prime }$.

In the recurrence for $WMB_{i,j}$ (Fig. 5), we distinguish two cases: (1) *j* is the right endpoint of a base pair $\left(j^{\prime },j\right)$ in *G*, and (2) *j* is unpaired in *G*. In the latter case, we ensure that *j* is paired in $G^{\prime }$ to close a pseudoloop; this case is handled by $WMB^{\prime }$.

In the first case of the *WMB* recurrence, $\left(j^{\prime },j\right)$ is a base pair of *G* and must be crossed by another pair $\left(k^{\prime },k\right)$ in $G^{\prime }$ to close the pseudoloop. Each choice of *k* defines a unique band in *G*, whose energy is computed by BE. Removing this band (closed by *j*) leaves (i) a prefix of the pseudoloop, whose energy is minimized by recursing to $WMB^{\prime }$, and (ii) a substructure spanning the region between *k* and the leftmost right border of the band around *j*. To optimize the energy of this latter region, HFold employs the recurrence *WI*, which, similarly to *W*, scores general unconstrained substructures, but in the context of the interior of a pseudoloop (using specific parameters).

**Figure 6:**
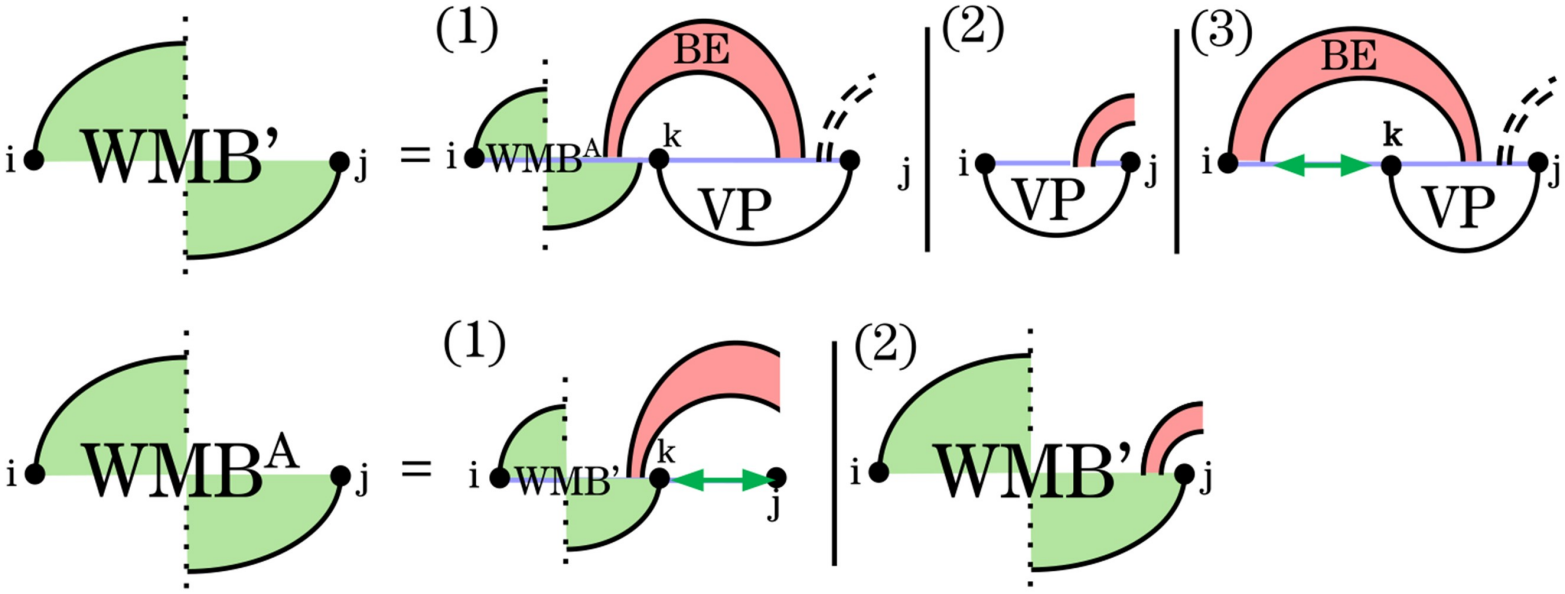
Non-sparse $WMB_{i,j}^{\prime }$ recurrence for pseudoloops and pseudoloop prefixes that do not end with a rightmost band in *G*. In Case 1, the two rightmost bands are removed, and the recurrence proceeds to pseudoloop prefixes with a potential recursive substructure on the right, which is handled by $WMB^{A}$. Cases 2 and 3 are terminal cases.

The recurrence $WMB^{\prime }$ (Fig. 6) covers all pseudoloops and pseudoloop prefixes in which *j* is paired in $G^{\prime }$. Unlike a full pseudoloop structure, a pseudoloop prefix is not closed, as it crosses base pairs of *G* that extend from the interval [i,j] to the right. Note that the energy of the crossing band is not included in the pseudoloop prefix energy.

Case 1 of $WMB^{\prime }$ resolves the two rightmost bands using the recurrences *BE* and *VP*, leaving a pseudoloop prefix and a potentially recursive substructure at the right end. To improve clarity relative to the original HFold recurrences, we handle these structures in an additional recurrence, $WMB^{A}$. The $WMB^{A}$ recurrence covers pseudoloop prefixes in which *j* is either unpaired (Case 1) or paired in $G^{\prime }$ (Case 2).

The recurrence *VP*, defined below, handles structures closed by a base pair of $G^{\prime }$ that crosses base pairs of *G*. Cases 2 and 3 of $WMB^{\prime }$ terminate the recursion by assigning the residual energy when only one or two bands remain in the pseudoloop.

**Figure 7:**
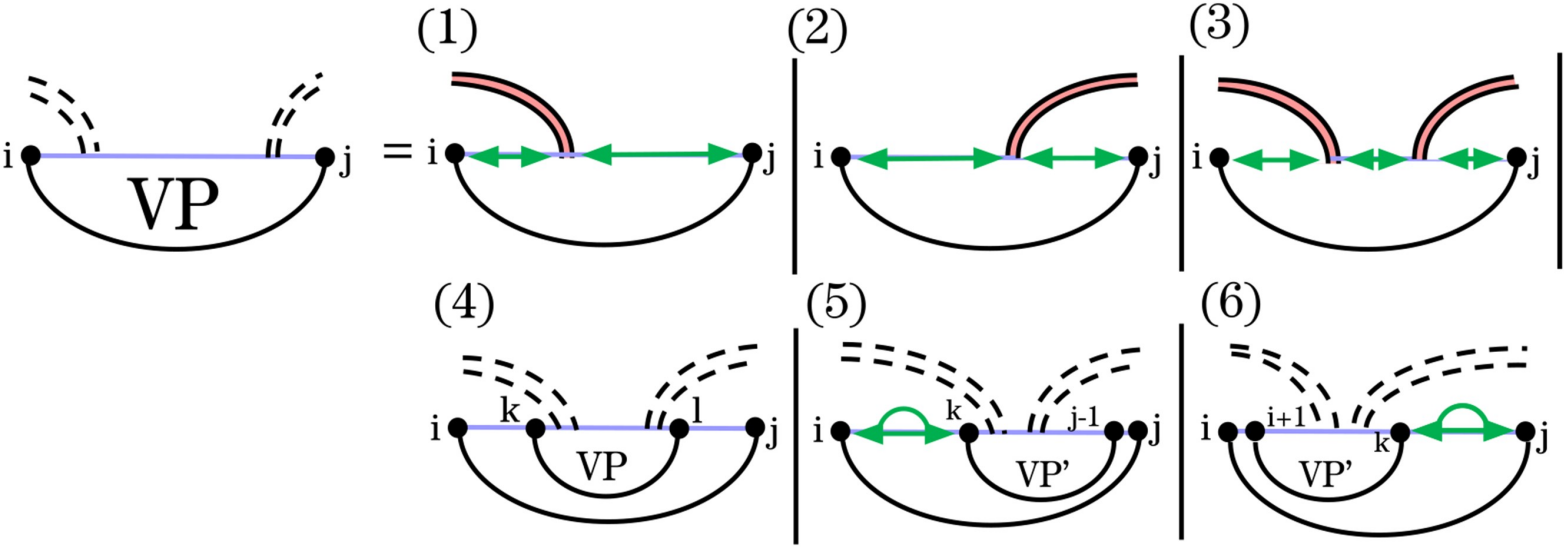
HFold’s $VP_{i,j}$ recurrence for structures in which the closing base pair, (i,j), crosses *G*. Cases 1–3 handle hairpins crossing base pairs of *G*; Case 4 handles interior loops, bulges, and stacks crossing *G*; Cases 5 and 6 handle multiloops that span a band. Recall the graphical notation from Fig. 3.

The recurrence for $VP_{i,j}$ (Fig. 7) handles substructures closed by a base pair (i,j) that crosses a band of *G*. We distinguish three types of loops closed by (i,j): hairpins (Cases 1–3), interior loops, bulges, and stacks (Case 4), and multiloops that span the band (Cases 5 and 6). These cases are further refined based on how (i,j) crosses the band(s) of *G*. To correctly score recursive structure in the context of multiloops that span a band, we introduce $WI^{\prime }$, in analogy to *W* and *WI*. In contrast to *W* and *WI*, $WI^{\prime }$ cannot correspond to an empty structure.

Cases 5 and 6 use $VP^{\prime }$  Supplementary Fig. 3 to ensure that multiloops contain at least one recursive base pair to the left or right of the band base pair. Moreover, this mechanism avoids simultaneous minimization over two indices.

## 3 The Spark algorithm

Recall that Spark and HFold solve the same problem, but Spark introduces sparsification to improve run-time and memory efficiency. Because Spark follows the general recursion scheme of HFold, we focus on describing the novelties introduced by sparsification and arguing their correctness. As outlined in the introduction, we developed Spark to achieve the same theoretical complexities as sparsified pk-free minimum free energy prediction (Will and Jabbari 2016). In particular, we replace the quadratic storage requirements of HFold with space only for sparse data structures (*Z* candidates and *T* trace arrows) and otherwise linear space. Notably, the space reduction achieved by sparsification goes hand in hand with an improvement in time complexity from $O(n^{3})$ to $O(n^{2}+nZ)$, since fewer entries need to be considered in the linear minimization cases.

We first outline the core sparsification strategy for pseudoknot-free MFE prediction and then extend it to pseudoknotted structures. Recall that $W_{i,j}$ is computed by minimizing over two alternatives: (i) nucleotides *i* and *j* close a loop, in which case the contribution is given by $V_{i,j}$; (ii) the optimal structure on the interval [i,j] can be split at some index *k* into two disjoint substructures, in which case we minimize over $W_{i,k-1}+W_{k,j}$ for all *k* with i<k≤j. The second minimization can be restricted to *candidates*. Here, $W_{k,j}$ corresponds to the energy of a closed structure (i.e. $W_{k,j}=V_{k,j}$), and there is no equal or better way to further decompose[k,j]. This restriction is justified because non-candidate splits cannot yield a better energy than candidate splits, due to the inequality $W_{i,j}\leq W_{i,k-1}+W_{k,j}$ (Wexler et al. 2007, Will and Jabbari 2016). Formally, [i,j] is a candidate if and only if it is not optimally decomposable, that is $V_{i,j}<W_{i,k-1}+W_{k,j}$ for all i<k≤j. Consequently, the minimization of $W_{i,k-1}+W_{k,j}$ is sparsified by changing it to


$$\min_{i<k<j,[k,j]\;\text{is}\;a\;\text{candidate}}W_{i,k-1}+V_{k,j}$$

and adding an extra case $W_{i,j-1}$ (to cover cases in which *j* is unpaired). In this way, we can efficiently evaluate the recurrences without storing the full quadratic dynamic programming matrices, as long as we retain the energies $V_{i,j}$ of the candidate intervals [i,j] in a sparse data structure.

**Figure 8:**
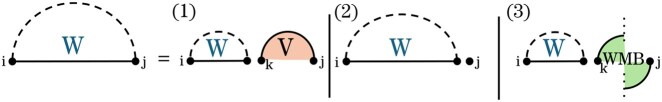
Revised *W* recurrence for sparsification. In Cases (1) and (3), the rightmost closed structure is removed, restricted to candidate substructures.

To handle pseudoknots, we introduce a third case, $WMB_{i,j}$, in which *i* and *j* close a pseudoloop. This yields the *W* recurrence shown in Fig. 8, which is an equivalent reformulation of HFold ’s original recurrence (Fig. 2) such that each case removes the rightmost closed substructure (or an unpaired base). In Case 1, a regular loop is removed; in Case 2, a single unpaired base; and in Case 3, a pseudoloop. We distinguish *V*-candidates from Case 1 and *WMB*-candidates from Case 3, and store them separately.

Notably, linear space suffices to store all required $W_{i,j}$ values in the dynamic programming of Spark. This is achieved by iterating over *i* in the outer loop (from right to left) and over *j* in the inner loop (from left to right), so that the linear space allocated for *W* entries can be reused for each new value of *i*. The same argument applies to all other required matrix entries that begin at *i* or within a constant bounded distance from *i*

This key idea enables computation of the MFE in sparse space but complicates the reconstruction of an optimal structure by traceback, since matrix entries are overwritten and only the candidates are stored persistently. For simple energy models, this problem was addressed by recomputation in Backofen et al. (2011).

When using a realistic energy model (such as HotKnots 2.0 or Turner 2004), recovering the MFE structure from candidates alone is not possible (Will and Jabbari 2016). To enable reconstruction in sparse space, we therefore maintain additional *trace arrows* that point to the optimal subproblems. These trace arrows are updated regularly and removed once they are no longer needed, using garbage collection (Will and Jabbari 2016).

In the following, we discuss the novel developments in Spark for sparsifying the decomposition of pseudoloops, leading to sparse analogues of *WMB*, $WMB^{\prime }$, $WMB^{A}$, *VP*, and auxiliary recurrences. We systematically rewrite the linear minimization cases of HFold to replace accesses to entries of the matrices *W*, *WI*, and $WI^{\prime }$ that do not originate at *i* or i+1 with candidates, as demonstrated for *W* in Fig. 8. While all such uses of *W* and even of $WI^{\prime }$ can be replaced in this way, this is not possible for all uses of *WI* in pseudoloops and within bands of $G^{\prime }$.

### 3.1 Linear data structures to hold *WI* entries

To maintain the non-replaceable *WI* entries in linear space, we introduce the novel data structures $WI^{R}$ and $WI^{L}$, indexed by sequence positions. The use of these arrays is motivated by the observation that all such *WI* entries are adjacent to base pairs of the given structure *G*. In $WI^{L}$ and $WI^{R}$, the left end of the *WI* region is determined by *G*, specifically by a left or right base-pair endpoint, respectively.

During Spark’s iteration over decreasing *i* (outer loop) and increasing *j* (inner loop), we store defined values $WI_{i,j}$

- at $WI_{j}^{L}$ if i−1 is the *left* end of a base pair in *G* and
- at $WI_{j}^{R}$ if i−1 is the *right* end of a base pair in *G*.

The correctness of Spark relies on loop invariants that hold after each iteration *i* of the outer loop. We say that a base pair (i,j)  *encloses* a position *k* if i<k<j. A region [i,j] is *weakly closed* if it is either closed or empty. Recall that $WI_{i,j}$ is defined only if [i,j] is weakly closed.

Lemma 1. *After iteration i*, $WI_{k}^{L}$  *equals* $WI_{\ell ,k}$*, where* (ℓ−1,r)  *is the smallest base pair of G that encloses k, if such a base pair exists*, i≤ℓ  *and* [ℓ,k]  *is weakly closed.*

Proof. If *k* is enclosed by some base pair of *G*, let (ℓ−1,r) be the smallest base pair of *G* that encloses *k*. By construction, the algorithm updates $WI_{k}^{L}$ only in those outer-loop iterations *i* and inner-loop iterations *j* for which

1. i−1 is the left endpoint of a base pair of *G* that encloses *k*, and
the region [i,j] is weakly closed, with j=k.

There is exactly one outer-loop index *i* that satisfies condition (1) together with the minimality of (ℓ−1,r), namely i=ℓ. In that iteration, [i,k]=[ℓ,k] is weakly closed by assumption, so the update condition holds and $WI_{k}^{L}$ is assigned the value $WI_{\ell ,k}$ in the inner-loop step j=k.

For any outer-loop iteration i≠ℓ, at least one of the two conditions above fails: either i−1 is not the left endpoint of the smallest enclosing base pair of *k*, or [i,j] is not weakly closed. Consequently, the update to $WI_{k}^{L}$ is skipped in all iterations with i≠ℓ. Thus, $WI_{k}^{L}$ is assigned exactly once, in iteration i=ℓ, and retains the value $WI_{\ell ,k}$, as claimed. □

While the opposite boundary of the region remains fixed in $WI^{L}$, the left boundary ℓ of $WI_{\ell ,r}$ (represented by $WI_{r}^{L}$) may decrease during the iteration over *i* in the case of $WI^{R}$.

Lemma 2. *After iteration i*, $WI_{r}^{R}$  *equals* $WI_{\ell ,r}$*, where* ℓ≥i  *is minimal among all base pairs* $\left(\ell^{\prime },\ell -1\right)$  *of G, provided such a base pair exists and* [ℓ,r]  *is weakly closed.*

Proof. The value $WI_{r}^{R}$ after iteration *i* is either undefined or was assigned in some outer-loop iteration $i^{\prime }$, where $\ell =i^{\prime }-1$ is the left endpoint of a base pair in *G* and, in the corresponding inner-loop iteration with j=r, the interval [ℓ,r] is weakly closed. Since the algorithm iterates over *i* in decreasing order, the current value of $WI_{r}^{R}$ after iteration *i* is precisely $WI_{\ell ,r}$ for the minimal ℓ≥i satisfying these conditions. □

### 3.2 Efficiently locating band borders in linear space

A further challenge is to determine the boundaries of bands in *G*. Whereas HFold uses quadratic tables of band boundaries to evaluate its recurrences efficiently, such tables would violate the sparse space constraints targeted by Spark. Instead, we locate outer band boundaries using range-minimum queries, which can be answered in constant time after linear-time preprocessing and require only linear space. Inner band boundaries can be obtained more easily by constant-time lookup in a linear array.

Concretely, the recurrences need to evaluate four band-boundary functions, *B*, $B^{\prime }$, *b*, and $b^{\prime }$ (see Supplementary Fig. 1). For a given region [i,j], $B^{\prime }(i,j)$ and B(i,j) specify, respectively, the outer and inner boundaries of the uniquely defined band that extends from [i,j] to the left, while b(i,j) and $b^{\prime }(i,j)$ give the corresponding outer and inner boundaries of the band extending to the right. All these functions return base-pair endpoints within the region [i,j]. Supplementary Section 1 provides further details and formal proofs.

### 3.3 Pseudoloops

**Figure 9:**
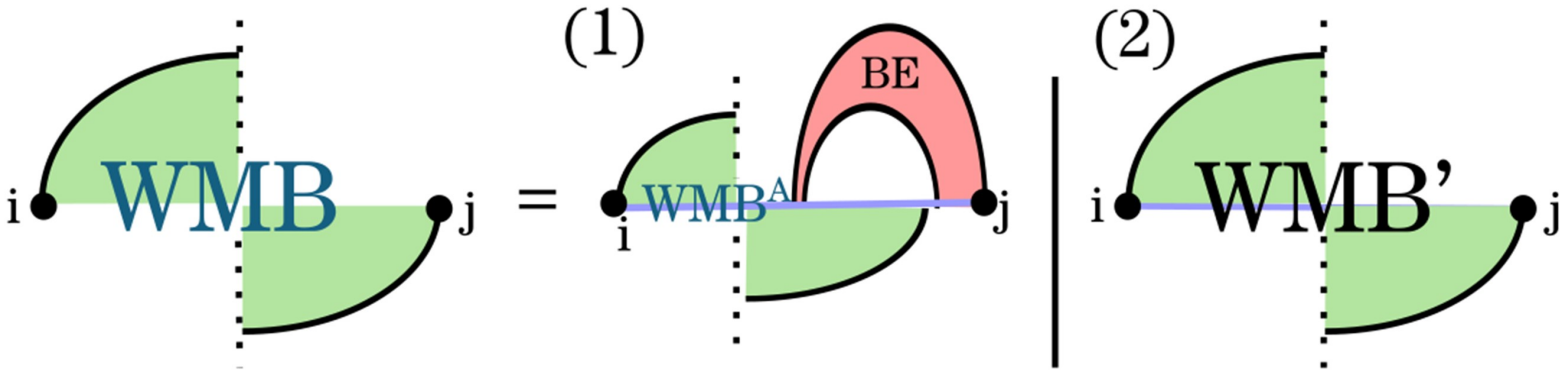
Rewritten recurrence of *WMB*. This equivalent recurrence avoids the use of *WI* and allows minimizing over BE candidates instead of linearly many *k*.

For Spark, we rewrite *WMB* (Fig. 9) to avoid minimization over linearly many values of *k*. Instead, the revised recurrences minimize only over BE-candidates. The equivalence to the original *WMB* recurrence of HFold is shown in the Supplementary Lemma 4.

**Figure 10:**
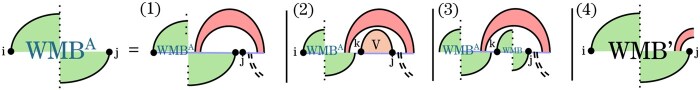
Rewritten $WMB^{A}$ recurrence. It directly decomposes the recursive substructure on the right. The cases successively consume, respectively, a single unpaired base, structures closed by regular loops, and pseudoloops, and finally allow an empty recursive substructure.

In the decomposition of pseudoloops and pseudoloop prefixes in $WMB^{A}$ (Fig. 6), we avoid using *WI* by expanding it into candidates and unpaired bases on the right. The modified recurrences are shown in Fig. 10.

Instead of recursing to *WI*, the revised $WMB^{A}$ recurrence successively eliminates the rightmost nested substructure by peeling off unpaired bases and closed substructures on the right. This allows us, in Cases 2 and 3, to restrict the minimization to candidate intervals for $V_{k,j}$ and $WMB_{k,j}$, which is essential for achieving the desired sparse time and space complexities.

The revised recurrence $WMB^{\prime }$ is mostly unchanged, apart from recursing to the revised $WMB^{A}$ (see Supplementary Lemma 5 for correctness). Notably, for space efficiency, the required *WI* entries in Case (3) are obtained as $WI_{k}^{L}$ due to Lemma 2.

### 3.4 Structures closed by a base pair crossing *G*

**Figure 11:**
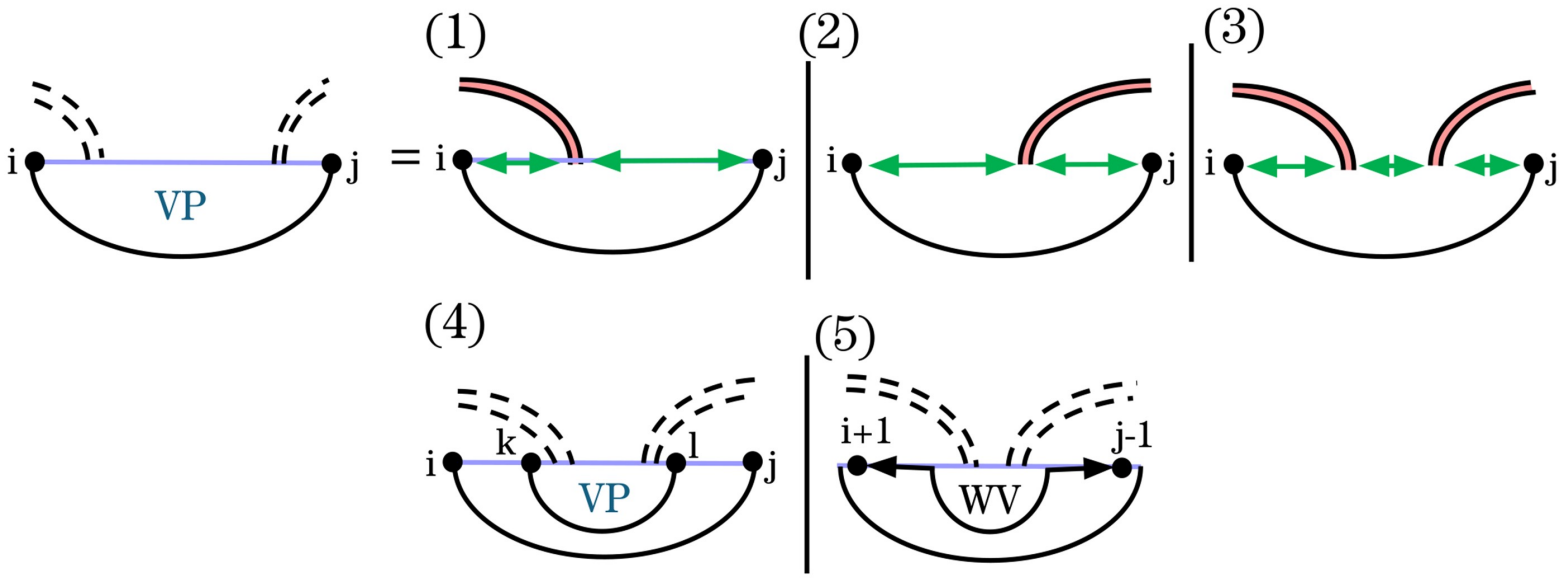
Updated *VP* recurrences for sparsification in graphical form. The revised *VP* cases are highlighted in blue.

Recall that in HFold, $VP_{i,j}$ minimizes over all structures $R_{i,j}$ in which $(i,j)\in G^{\prime }$ crosses at least one base pair in *G* (Fig. 7). If no such structure exists, then VP(i,j)=∞. The revised recurrences used in Spark are illustrated in Fig. 11.

### 3.5 Band termination in *VP*

Cases 1–3 of the updated *VP* follow the logic of the original *VP*. Here, (i,j) is the innermost base pair of a band, corresponding to a hairpin. These cases allow recursive substructures between the crossing bands of *G* without exceeding linear space.

Lemma 3 *All required WI in VP can be provided in linear space.*

Proof. All *WI* entries originating at i+1 are explicitly stored by Spark and overwritten in each new iteration of the outer loop, following a standard strategy in sparse folding algorithms. The remaining required *WI* energies are obtained via the new $WI^{L}$ and $WI^{R}$ arrays. Specifically, in Case 1 we read the appropriate *WI* value as $WI_{j-1}^{R}$; in Case 2, as $WI_{j-1}^{L}$; and in Case 3, we access the middle *WI* value as $WI_{r-1}^{R}$, where *r* is the corresponding band boundary in *G*, and the right *WI* value as $WI_{j-1}^{L}$. The correctness of these accesses follows from the definition of the recurrence, which satisfies the exact conditions of Lemmas 2 and 1. □

### 3.6 Interior loops in *VP*

While Case 4 is unchanged in the graphical notation, its implementation in Spark is adapted analogously to the treatment of *V* in previous sparse folding algorithms (Will and Jabbari 2016, Gray et al. 2024). Specifically, the minimization is optimized by considering *VP*-candidates first, and trace arrows to the optimal inner base pair are retained to enable structure reconstruction during traceback.

### 3.7 Multiloops in *VP*

In Case 5 of *VP* in Spark, (i,j) closes a multiloop that spans a band. In such multiloops, one inner base pair crosses the same band(s) of *G* as (i,j), while the remaining bands and unpaired bases are treated as nested substructures via the $WI^{\prime }$ recurrence. The original Cases 5 and 6 (Fig. 7) decomposed these structures by either breaking off a non-empty region on the left or right through $WI^{\prime }$ and decomposing the remainder using the $VP^{\prime }$ recurrence (Fig. 3). To support sparsification, we transform these multiloop cases using the novel auxiliary recurrences *WV* and $WV^{e}$.

### 3.8 Novel auxiliary recurrences *WV* and $WV^{e}$ to decompose multiloops

**Figure 12:**
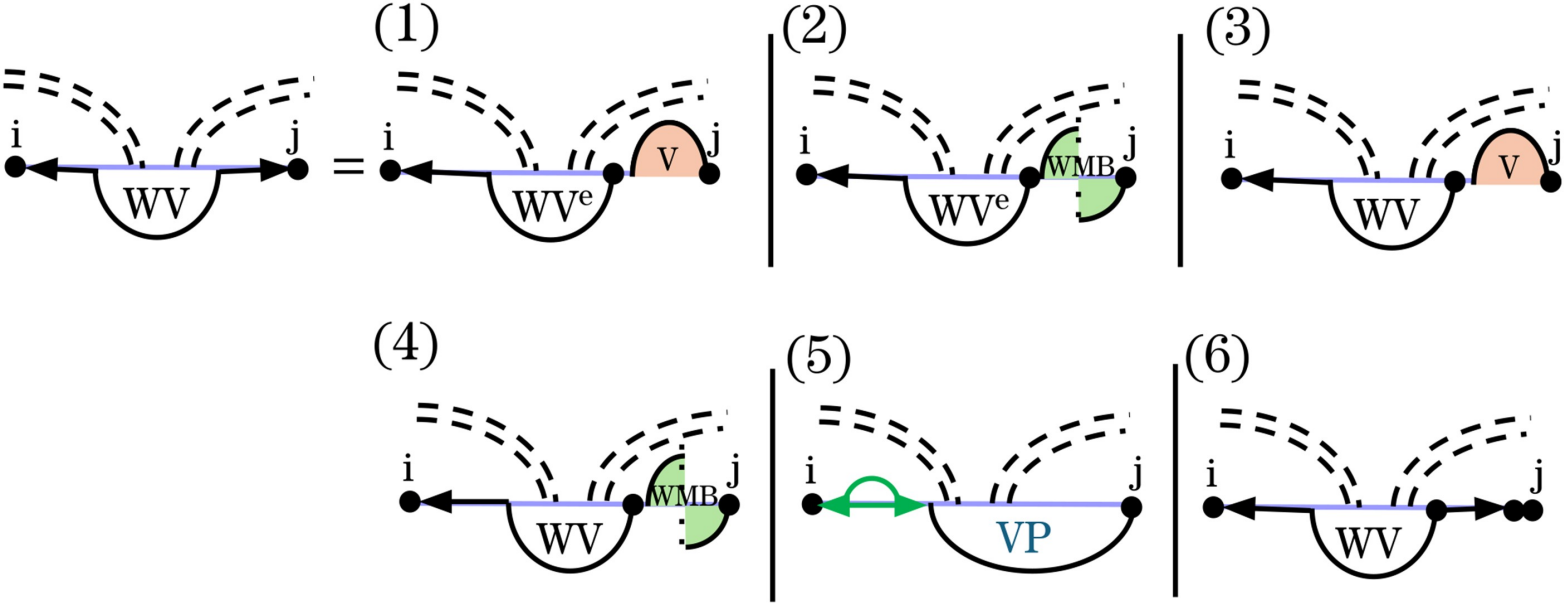
Graphical representation of the *WV* recurrences illustrating how a multiloop that spans a band is decomposed. In Cases 1 and 2, the left region is empty. In Case 1, *j* pairs with a base in the right region of the band, forming a regular loop, whereas in Case 2, *j* is the closing base of a pseudoloop. Cases 3, 4, and 6 iteratively remove the rightmost substructure (or leave an empty region in Case 6) and recurse back to *WV*. Case 5 processes the leftmost nested substructure within a pseudoloop and recurses to *VP*.

We define *WV* to handle the interior of multiloops that span a band (Fig. 12). Formally, $WV_{i,j}$ is the MFE of all valid structures $R_{i,j}$ over the region[i,j], under the conditions that (i) [i,j] is not weakly closed (i.e. at least one base in the region pairs with a base outside the region), and (ii) [i,j] contains at least two inner base pairs, one of which forms a *VP* structure.

To enforce the latter property, we introduce an additional auxiliary recurrence, $WV^{e}$. Similar to *WV*, $WV^{e}$ handles parts of a band-spanning multiloop but does not allow any non-empty recursive substructure on either side of *VP*.

### 3.9 Single cases of *WV* and $WV^{e}$

Cases 1–4 (Fig. 12) reduce *WV* by removing a closed structure *V* or *WMB* at the right end. Since these cases guarantee a non-empty substructure to the right of *VP*, they either terminate the *WV* recursion by recursing to $WV^{e}$ (Cases 1 and 2) or continue the recursion (Cases 3 and 4). In other words, Cases 1–4 collectively cover all admissible configurations with structure on the right and either empty or non-empty structure on the left. In all of these cases, the minimization is restricted to candidates.

In Case 5, the rightmost structure is a *VP*-candidate. Notably, the remaining $WI^{\prime }$ structure to the left of *VP* is guaranteed to be non-empty. In the remaining Case 6, a single unpaired base at position *j* is removed.

**Figure 13:**
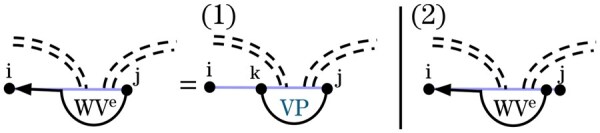
Graphical representation of $WV^{e}$ recurrence. In Case 1 *j* pairs with *k* forming a *VP* structure and region [i,k] is empty, and in Case 2 *j* is unpaired.

Figure 13 illustrates the $WV^{e}$ recurrence. It decomposes $WV^{e}$ structures by removing *VP* substructures or unpaired bases on the right. Notably, Case 1 is sparse, that is it is restricted to *VP*-candidates.

## 4 Constant time retrieval of BE candidates

Recall that Spark computes BE entries (see recurrences in Supplementary Section 5.14) within its main loop, iterating over decreasing *i* and, for each *i*, increasing j>i.

This computation order yields a technical lemma that enables constant-time retrieval of the required BE entries, both within the BE computation itself and in the evaluation of $WMB^{\prime }$.

Lemma 4. *At outer iteration i and for any* j>i*, if the candidate list CLBE[j] has last entry* (ℓ,e)  *with* ℓ>i*, then* ℓ  *is the minimal index satisfying* ℓ>i  *such that* $\left(\ell ,{\mathrm{bp}}_{G}(j),j,{\mathrm{bp}}_{G}(\ell )\right)$  *is a BE-candidate.*

### 4.1 Proof sketch

At iteration i,j, Spark evaluates *BE* at $\left(i,{\mathrm{bp}}_{G}(j),j,{\mathrm{bp}}_{G}(i)\right)$, obtaining a band energy *e*. If this tuple is a BE-candidate, Spark appends the pair (i,e) to the candidate list *CLBE[j]*.

Because the outer loop iterates over *i* in decreasing order, the BE-candidates $\left(i,{\mathrm{bp}}_{G}(j),j,{\mathrm{bp}}_{G}(i)\right)$ are appended to *CLBE[j]* in strictly decreasing order of *i*. Hence, among all indices ℓ>i for which $\left(\ell ,{\mathrm{bp}}_{G}(j),j,{\mathrm{bp}}_{G}(\ell )\right)$ is a BE-candidate, the last entry (ℓ,e) in *CLBE[j]* corresponds to the minimal such ℓ, as claimed. □

Corollary 5. *Each BE entry can be computed by Spark in constant time. Moreover, in the* $WMB^{\prime }$  *recurrence, the required BE entries can also be retrieved by Spark in constant time.*

### 4.2 Proof sketch

Lemma 4 implies that the enclosing BE-candidate needed in the BE recurrence can be located in constant time as the last entry of the corresponding candidate list. Similarly, in $WMB^{\prime }$, the correct band position and its energy are obtained in constant time from the appropriate BE candidate list. The indices of these lists are determined by the left inner band boundaries (Section 3.2). □

### 4.3 Time and space complexity

After sparsification, Spark achieves efficient bounds in both time and space. Recall that *n* denote the sequence length, *Z* the total number of candidates due to sparsification, and *T* the number of trace arrows stored for back-tracing the MFE structure.

Theorem 6. *Spark runs in* $O(n^{2}+nZ)$  *time and* O(n+Z+T)  *space.*

The time and space complexities of Spark, which parallel those of previous sparse pseudoknot-free energy minimization algorithms, arise from general sparsification strategies combined with careful rewriting, adaptation, and optimization to meet Spark-specific requirements–most notably, the storage and retrieval of *WI* and, in particular, BE entries in the recurrences for $WMB^{\prime }$ and BE. We detail the sparse time and space requirements of Spark separately below.

**Sparse time complexity:** The sparse time complexity results from iterating over $O(n^{2})$ pairs (i,j) and minimizing over candidates. The accumulated total number of minimization over candidates in every steps per outer iteration is *Z*. As it is common practice for structure prediction implementations, the size of interior loops are limited, such that the minimizations over all possible interior loops in *V*, and notably also *VP* take constant time.

**Sparse space complexity:** The memory bounds of Spark are determined by several components that depend on the input sequence and structure:

1. A linear data structure used to locate band borders in constant time via range-minimum queries.
2. *Linear DP tables*. During dynamic programming, with iteration over *i* in the outer loop, we store all solutions to subproblems that start at *i* (or within a constant offset of *i*) in tables of linear size. This technique is well known from previous space-sparse folding algorithms. Notably, bounding the size of interior loops (whether crossing or non-crossing) limits the space for *VP* analogously to *V*.
3. Linear data structures storing *WI*.
4. *Candidates*. Due to sparsification, apart from linear DP tables, solutions to subproblems are stored only for candidate intervals, so the corresponding space is bounded by their total number *Z*.
5. *Trace arrows* required for recovering optimal structures during traceback, with space bounded by their number *T*. As in previous work Will and Jabbari (2016), *T* is further reduced by garbage collection.

## 5 Results

### 5.1 Benchmarking data and experimental design

#### 5.1.1 Benchmarked prediction tools

We empirically evaluated the time and memory requirements of Spark, comparing it to RNAfold, HFold, SparseRNAFolD, LinearFold, and Knotty. These tools were selected to provide distinct reference points and to address specific questions:

- HFold is the most direct reference, as it implements a non-sparse algorithm that solves exactly the same folding problem as Spark.
- The highly optimized, pseudoknot-free RNAfold serves as a lower bound on realistically achievable folding performance without sparsification.
- SparseRNAFolD and LinearFold both implement performance-enhanced, pseudoknot-free folding algorithms. While the beam-pruning heuristic of LinearFold sacrifices optimality, the sparsification in SparseRNAFolD preserves exactness and guarantees optimal predictions.
- Knotty provides an additional point of comparison for Spark, since it is sparsified in the same sense as Spark and SparseRNAFolD, yet covers a comparably rich class of pseudoknots in a direct, non-hierarchical folding framework.

#### 5.1.2 RNAstrand-based benchmark RNAs

We primarily benchmarked Spark on the dataset from SparseRNAFolD (Will and Jabbari 2016), which consists of 3704 RNA sequences grouped into six families and sourced from the RNAstrand V2.0 database (Andronescu et al. 2008). Sequence lengths range from 8 to 4381 nucleotides.

#### 5.1.3 Long virus RNA (SARS-CoV-2)

To expand the dataset and evaluate longer sequences, we additionally included the SARS-CoV-2 genome (length 29 903 nucleotides) with a constraint structure derived by Ziesel and Jabbari (2024). This constraint structure was obtained by combining all non-overlapping stems into a minimal free-energy constraint structure. Furthermore, to systematically examine the effect of sequence length, we extracted partial segments of the SARS-CoV-2 genome, starting at length 4000 nucleotides and increasing in steps of 2000 nucleotides up to the full-length genome.

#### 5.1.4 Generation of input structures

Constraint structures were generated by randomly selecting pairs of indices within each sequence. If the chosen bases could pair and were separated by at least three nucleotides, the base pair was extended via stacking interactions to form the maximal stem. Stem energies were calculated as sums over their single stacking energies. The stem with the lowest energy among all stems was selected as the constraint structure. This strategy permits to select potential long-range interactions, including multiloops or pseudoknots.

Because constrained pseudoknot prediction requires an input structure scaffold, all algorithms–including RNAfold–received the same input structure for each sequence. For all sequences, we selected the minimum-free-energy stem–loop as described in Section 5.1, ensuring a fair comparison.

#### 5.1.5 Hardware and performance measures

All experiments were run on an M2 Max Mac Studio with 64 GB of RAM. Runtimes were recorded as user CPU time and memory usage as maximum resident set size.

### 5.2 Spark and HFold predict identical energies

Spark’s implementation was validated against HFold on our dataset. Spark and HFold predicted identical MFE values across all test cases. As expected, since the optimal structures are not necessarily unique, the tools report different optimal structures in few cases. Detailed results of this comparison are published online together with the software.

### 5.3 Spark outperforms HFold and closely matches RNAfold

Remarkably, despite predicting more complex pseudoknotted structures, Spark–highlighting the benefits of sparsification– achieves running times that are very close to those of RNAfold, and even improves on RNAfold in terms of memory usage. In contrast, the comparison with HFold [as implemented in Jabbari et al. (2008)] more directly isolates the effect of sparsification and local optimizations, since HFold and Spark solve exactly the same pseudoknotted folding problem.

Because HFold can process only sequences shorter than 5000 nt, it was evaluated solely on the original SparseRNAFolD dataset (Gray et al. 2024). On this set, HFold ’s maximum runtime and memory footprint were 31,047 s and 937 MB, respectively. By comparison, RNAfold required at most 12.8 s and 106 MB, while Spark needed only 15.7 s and 35.8 MB.

### 5.4 Spark non-heuristically predicts potentially pseudoknotted SARS-CoV-2 genome structure

The performance gains from sparsification can be expected to enable folding of substantially longer sequences. Even for pseudoknot-free folding, the SARS-CoV-2 genome is challenging. For example, LinearFold has been reported to fold this genome rapidly without heuristic restrictions beyond beam pruning (cf. our observations below). By contrast, SparseRNAFolD, while exact, required a still moderate 514.61 s and 263 MB due to sparsification. RNAfold, however, demanded considerably more resources in our benchmark, namely 2927 s and 4.81 GB.

**Figure 14:**
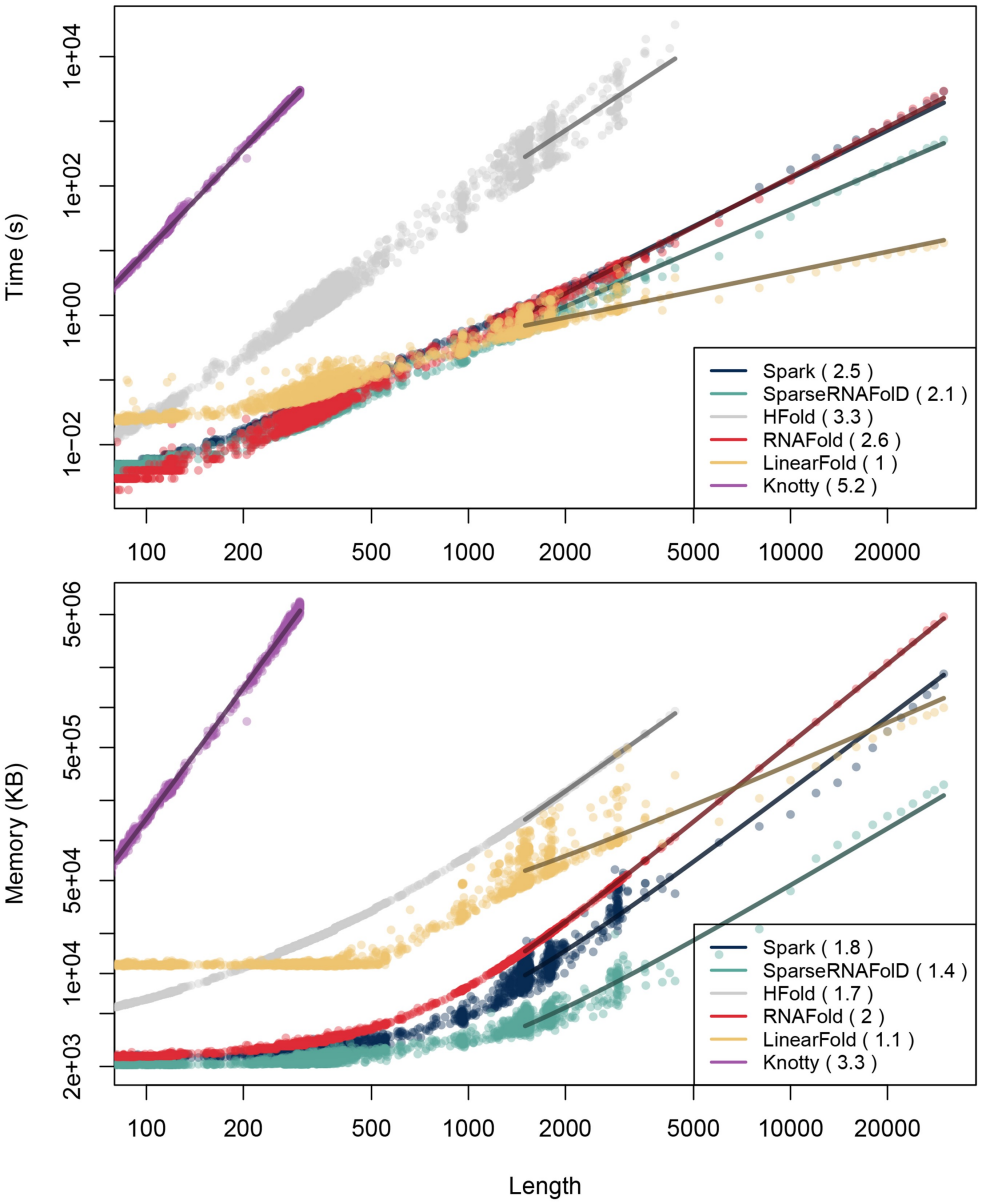
Performance of Spark and five state-of-the-art algorithms: SparseRNAFolD, HFold, RNAfold, LinearFold and Knotty. All tools (except Knotty) were run on all sequences with structure constraints from our benchmark dataset. Run time and memory usage are shown as functions of input length (respective log–log plots on top and bottom). Asymptotic behavior was estimated by parameter fitting for lengths n≥2000 (for Knotty, n≥50). The fitted trends are shown as solid lines, and the corresponding exponents *x* are reported in parentheses in the legend, facilitating direct comparison of empirical complexities $\Theta (n^{x})$.

For pseudoknot-prediction algorithms, the full SARS-CoV-2 genome (29 903, nt) lies far beyond the practical range of HFold, whereas Spark folds the entire genome without any additional heuristic restrictions in 2869 s using 1.43 GB. Results across a range of sequence lengths are summarized in Fig. 14.

#### 5.4.1 Constraint-dependent performance of LinearFold

Remarkably, we observe a substantial impact of the input constraint structure on LinearFold’s memory consumption. When the constraint includes long-range interactions, LinearFold is forced to retain these long-range dependencies, which limits its ability to store data sparsely.

With a constraint structure containing only short-range interactions, LinearFold required 13.18 s and 996 MB on the SARS-CoV-2 sequence. In contrast, when provided with a long-range interaction (a stem spanning nearly the entire sequence), the time and space usage increased to 715.38 s and 32 102 MB, respectively—an increase by a factor of approximately 55 in time and 32 in space. This highlights the importance of understanding how input constraints influence the time and space behavior of sparsified algorithms.

### 5.5 Knotty as sparsified out-of-competition reference

The comparison with Knotty highlights the fundamental differences between hierarchical and non-hierarchical folding. In stark contrast to the sparsified hierarchical folding algorithm Spark, the likewise sparsified but non-hierarchical pseudoknot prediction tool Knotty is limited to sequences of at most 400 nt due to its memory requirements. In our benchmark, the largest sequence that Knotty could fold had length 300 nt, for which it still required 3028 s and 6.2 GB of memory.

### 5.6 Effect of input constraints on time and space

To assess how Spark’s performance is affected by the input structures, we systematically generated combinations of random sequences and constraint structures. We generated 100 sequences of each length 1000, 5000 and 10 000 and generated 22 constraint structures per sequence in different ways: we chose the 20 energetically best Hotspots (i.e. single stems, see Supplementary Alg. 1), the RNAfold MFE structure, and one structure by greedily selecting log(n) best non-overlapping Hotspots.

The results show substantial variation in performance across sequences and input structures of the same length (see Supplementary Fig. 4). This variation reflects differences in the effectiveness of sparsification—note that memory consumption is directly tied to the number of candidates and trace arrows.

### 5.7 Number of candidates in pseudoknotted vs. pk-free folding

We counted *V*-candidates and trace arrows in Spark and compared them to the corresponding quantities in a pk-free variant of Spark (pseudoknots disabled). In this direct comparison within the same tool, we observe only a modest increase in both counts due to pseudoknots (Supplementary Fig. 5).

This effect arises from the energy parameters. Switching to pseudoknot recurrences introduces stronger penalties for unpaired bases inside pseudoloops, making base pairs that reduce the number of unpaired bases energetically more favorable. Consequently, additional *V*-candidates and trace arrows must be retained, because they participate in *WI* and $WI^{\prime }$ computations [see Gray et al. (2024) for analogous rules in the pk-free case].

Empirically, the growth remains moderate: on our largest sequences, we observe at most a four-fold increase in *V*-candidates and a two-fold increase in *V* trace arrows relative to the pk-free setting. For all sequence lengths, the numbers of candidates and trace arrows remain far below the number of entries in the corresponding non-sparse matrices. We furthermore observe that the total number of candidates and trace arrows grows more slowly than a quadratic function.

## 6 Conclusion

Like its predecessor HFold, our novel algorithm Spark computes pseudoknotted RNA structures that optimally extend given pseudoknot-free secondary structures. This form of constrained pseudoknot prediction is directly motivated by the hierarchical folding hypothesis of RNA structure formation.

Compared with previous RNA pseudoknot prediction algorithms, including HFold, Spark substantially improves time and memory requirements through the use of sparsification techniques. This yields a highly efficient tool for pseudoknotted folding under a realistic energy model (HotKnots 2.0).

Algorithmically, Spark performs exact energy minimization by efficiently solving a system of recurrences using sparse dynamic programming. This leads to two key characteristics of our hierarchical pseudoknot prediction approach: (i) In contrast to heuristic RNA folding methods, Spark guarantees the optimality of its predictions. (ii) Spark ’s asymptotic time and space complexity bounds match those of previous pseudoknot-free sparse energy minimization algorithms (e.g. SparseRNAFolD). In particular, sparsification in Spark reduces cubic time in the sequence length *n* to a running time on the order of $n^{2}$ plus *n* times the number of candidates, and reduces quadratic space to linear space plus the storage required for candidates and trace arrows.

We emphasize that achieving these complexity bounds in Spark required several novel developments and techniques for RNA energy minimization. Specifically, we introduced new data structures, employed range-minimum queries, defined a new pseudoknot-specific (pk-specific) type of candidate, and formally proved its correctness.

We provide a carefully optimized C++ implementation that uses the ViennaRNA library for elementary energy contributions. The tool was validated and extensively benchmarked against other MFE folding tools, including RNAfold, HFold, LinearFold, SparseRNAFolD, and Knotty. On a benchmark where HFold required 31 047 s and 937 MB, Spark finished in only 15.7 s while using 35.8 MB of memory, corresponding to improvements by factors of almost 2000 in time and 26 in space. Spark can fold long genomes of RNA viruses such as SARS-CoV-2 (approximately 30 000 nt), which are well beyond the reach of HFold; in this setting, it even outperforms the pk-free RNAfold.

Spark ’s capabilities open the door to routine pseudoknot-aware large-scale analyses, for example of large viral genomes or transcriptome-wide studies, while preserving the optimality guarantees of exact dynamic programming and avoiding ad hoc or weakly controlled heuristic restrictions.

## Supplementary Material

btag194_Supplementary_Data

## Data Availability

Spark software is available on Github (https://github.com/TheCOBRALab/Spark), with a permanent archive of the software and results deposited on Zenodo (https://doi.org/10.5281/zenodo.19073315).
