# Supplementary material for: Spark: sparse hierarchical energy minimization for scalable prediction of RNA pseudoknots
Source: Bioinformatics. 2026 Apr 21;42(5):btag194. doi: 10.1093/bioinformatics/btag194 (PMC13171173; doi:10.1093/bioinformatics/btag194)
Supplement: btag194_Supplementary_Data [file btag194_supplementary_data.pdf]

Supplemental Material
Spark: Sparse Hierarchical Energy Minimization for Scalable Prediction of RNA Pseudoknots

Mateo Gray , Sebastian Will and Hosna Jabbari

1 Band boundaries

Let G denote an RNA structure. $\text{bp}_G(i)$ denotes the pairing partner of i in G, i.e.

$$\text{bp}_G(i) = \begin{cases} j & \text{if } (i,j) \in G \text{ or } (j,i) \in G \\ -1 & \text{otherwise} \end{cases}$$

For positions i and j, $i \le j$, $B'(i,j), B(i,j), b(i,j)$ and $b'(i,j)$ respectively denote left and right-most boundaries in $[i,j]$ of the maximal G-bands extending from $[i,j]$ to the right or left (Fig. 1). Formally, we define

$$\begin{aligned} B'(i,j) &:= \min\{r \mid \ell < i \le r \le j, (\ell,r) \in G\} \cup \{\infty\} \\ B(i,j) &:= \max\{r \mid \ell < i \le r \le j, (\ell,r) \in G\} \cup \{-1\} \\ b(i,j) &:= \min\{\ell \mid i \le \ell \le j < r, (\ell,r) \in G\} \cup \{\infty\} \\ b'(i,j) &:= \max\{\ell \mid i \le \ell \le j < r, (\ell,r) \in G\} \cup \{-1\} \end{aligned}$$

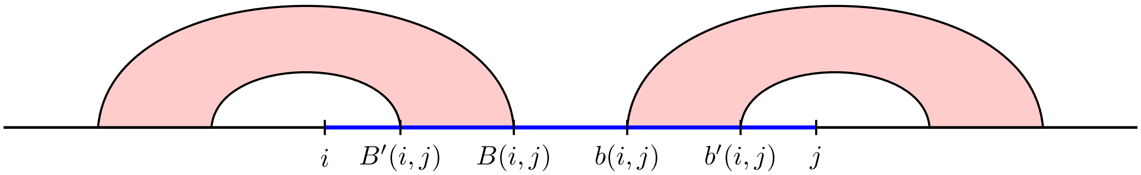

Figure 1: Band boundaries.

Definition 1. *A base pair $(i,j) \in G$* left encloses *a position k in G, iff $i < k \le j$. It* directly left encloses *k iff there is also no other base pair $(i',j') \in G$, such that $i < i' < k \le j' < j$.*

Lemma 1. *If there is a base pair $(\ell,r) \in G$ that directly left encloses i and $r \le j$ then $B'(i,j)$ equals r; otherwise ∞.*

Proof. By definition $B'(i,j)$ is the minimum right end of any base pair (ℓ,r) in G, where $\ell < i \le r \le j$, if such a base pair exists; it is ∞ otherwise. Assume that such a base pair exists, then it left encloses i. It also directly left encloses i, since $(i',j') \in G$, such that $\ell < i' < k \le j' < r$, would left enclose i and violate the minimality of r due to $j' < r$. □

Definition 2.

$$\text{bp}^+_G(i) = \begin{cases} \text{bp}_G(i) & \text{if } \text{bp}_G(i) \ge 0 \\ \infty & \text{otherwise} \end{cases}.$$

Lemma 2. *For sequence positions i and j, let $\ell^* = \min_{i \le r \le j} \text{bp}^+_G(r)$. Then,*

$$B(i,j) = \begin{cases} \text{bp}_G(\ell^*) & \text{if } \ell^* < i \\ -1 & \text{otherwise} \end{cases}$$

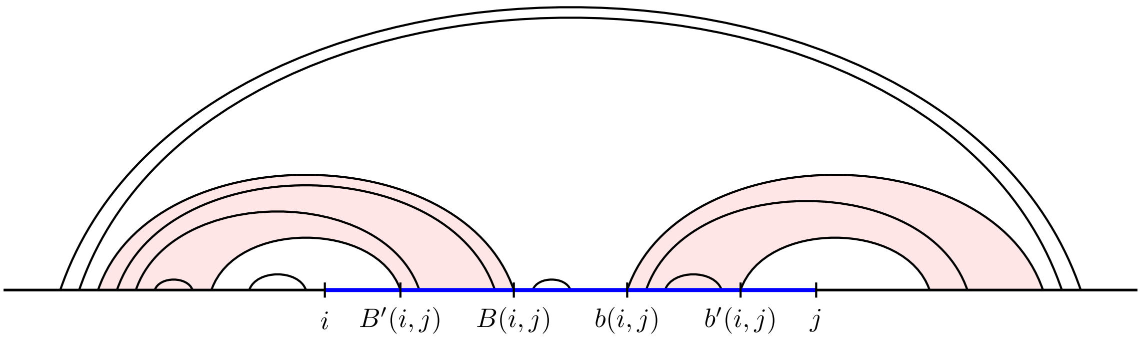

Figure 2: Example for band boundaries.

Proof. By definition $B(i,j)$ is the largest right end of a base pair $(\ell, r) \in G$, where $\ell < i \leq r \leq j$. First, assume that there is such a base pair. Then, $\ell^* < i$ and $(\ell^*, \mathrm{bp}_G(\ell^*))$ is a base pair of G, where $\ell^* < i \leq \mathrm{bp}_G(\ell^*) \leq j$. ℓ^* is the minimum left end, such that $\mathrm{bp}_G(\ell^*)$ is the maximum right end of any such base pair since G is non-crossing. If and only if there is no such base pair $(\ell, r) \in G$ then $\ell^* \geq i$, such that the Lemma correctly yields -1. □

Lemma 3. *All band boundary functions can be processed in constant time per query and linear space.*

Proof. Due to symmetry, it suffices to show the claim for B and B'.

$\boldsymbol{B'(i,j)}$**.** To retrieve B' in constant time, a linear array of size n to find directly left enclosing base pairs can be precomputed in linear time and space by traversing the base pairs of G.

$\boldsymbol{B(i,j)}$**.** We observe that $\min_{i \leq r \leq j} \mathrm{bp}^+_G(r)$ has the form of a range minimum query (RMQ). RMQs can be answered in constant time with linear space requirements after linear time precomputation [Fischer and Heun, 2007]. □

2 Definitions

- **RNA molecule**: A sequence of nucleotides, or bases, of length n, of which there are four types: Adenine (A), Guanine (G), Cytosine (C), and Uracil (U).
- **Base pair**: When a RNA folds, bonds form between the bases of the molecule, where each base may pair with at most one other base.
- **RNA structure R**: The set of base pairs (i, j), $1 \leq i < j \leq n$ such that no index occurs in more than one base pair, and each base pair is one of the canonical base pairs: $\{A - U, C - G, G - U\}$.
- $\mathrm{bp}_{\mathbf{R}}(\mathbf{i})$: We let $\mathrm{bp}_R(i)$ denote the index of the base that is paired with base i in R, if any.
- **cross**: if $(i, j), (i', j')$, and $i < i' < j < j'$, we say that the pair (i, j) crosses the pair (i', j'); and vice versa, (i', j') crosses (i, j).
- **Pseudoknotted base pair**: We say that (i, j) is a pseudoknotted base pair if for some other base pair (i', j') in R, (i, j) crosses (i', j').
- **Pseudoknot-free structure**: If there are no pseudoknotted base pairs in the given structure, it is called a pseudoknot-free (pk-free) secondary structure.
- **Cover**: Let G be a pk-free structure. Base pair (i, j) *covers* base k if $i < k < j$ and there is no other base pair (i', j') where $i < i' < k < j' < j$.
- **is_covered**$_G(k)$: true iff some base pair of G covers k.
- **Region** $[i, j]$: Sequence of indices between i and j inclusive.
- **Disjoint region**: two regions $[i, j]$ and $[i', j']$ are disjoint if no index is in both regions, i.e $j < i'$ or $j' < i$
- **Weakly closed region**: A region is weakly closed if no base pair of G connects a base in the region to a base outside the region.
- **Closed region**: A weakly closed region $[i, j]$ with at least two bases is closed, if it cannot be partitioned into two independent smaller weakly closed regions. Note that if $[i, j]$ is closed, then both i and j must be paired, although not necessarily with each other [Rastegari and Condon, 2007].
- **Pseudoknotted closed region**: a closed region $[i, j]$ of a structure R such that $i.\mathrm{bp}_R(i)$ and $\mathrm{bp}_R(j).j$ are pseudoknotted base pairs.
- **directly banded in**: For a pseudoknotted base pair (i, j), we say (i, j) is *directly banded in* (i', j'), denoted $(i, j) \preceq (i', j')$, if $i' \leq i < j \leq j'$ and $[i' + 1, i - 1]$ and $[j + 1, j' - 1]$ are weakly closed regions
- **Band**: Consider a maximal chain of $\preceq$. The minimum (maximum) base pair in the maximal chain is the band's inner (outer) closing pair. If (i, j) is the outer and (i', j') the inner closing pair of a band, then $[i, i']$ and $[j', j]$ are the band's regions
- **Pseudoloop**: Let $[i, j]$ be a pseudoknotted closed region. Then the unpaired bases and base pairs associated with $[i, j]$, together with the closing base pairs of the band associated with $[i, j]$, are members of a *pseudoloop.* The base pairs $(i, \mathrm{bp}_R(i))$ and $\mathrm{bp}_R(j).j$ are the closing base pairs of the pseudoloop.
- **Bi-secondary structure**: A structure R that can be formed by the union of two disjoint pk-free secondary structures [Witwer et al., 2004].
- **Density**: We define density as follows: Let L be a pseudoloop and $i.\mathrm{bp}_R(i)$ and $\mathrm{bp}_R(j).j$ be the closing base pairs of L. Let #B(L,k) be the number of bands associated with L that cross k. Then the density of L is the max #B(L,k) for all k in region $[i, j]$. The density of a structure, R, is the maximum density of L over all pseudoloops L of R. We say R is a density-2 structure if the density of R is at most 2.

3 Energy Model

Table 1: ENERGY PARAMETERS. All parameters were derived at 37 degrees celsius and 1 M salt (NaCl) concentration or extrapolated from experimental values cf. Jabbari et al. [2008], Andronescu et al. [2010], Ren et al. [2005].

Name	*Description*	*Value (kcal/mol)*
P_s	Exterior pseudoloop initiation penalty	-1.38
P_{sm}	Penalty for introducing pseudoknot inside a multiloop	10.07
P_{sp}	Penalty for introduce pseudoknot inside a pseudoloop	15.00
P_b	Band penalty	2.46
P_{up}	Penalty for unpaired base in a pseudoloop	0.06
P_{ps}	Penalty for closed subregion inside a pseudoloop	0.96
$e_H(i,j)$	Energy of a hairpin loop closed by (i,j)	
$e_S(i,i+1,j-1,j)$	Energy of a stacked pair closed by (i,j)	
$e_{stP}(i,i+1,j-1,j)$	Energy of a stacked pair that spans a band	$0.89 \times e_S(i,j)$
$e_{int}(i,r,r',j)$	Energy of a pk-free internal loop	
$e_{intP}(i,r,r',j)$	Energy of an internal loop that spans a band	$0.74 \times e_{int}(i,d,e,j)$
a	Multiloop initiation penalty	3.39
b	Multiloop base pair penalty	0.03
c	Penalty for unpaired base in a multiloop	0.02
a'	Penalty for introducing a multiloop that spans a band	3.41
b'	Base pair penalty for a multiloop that spans a band	0.56
c'	Penalty for unpaired base in a multiloop that spans a band	0.12

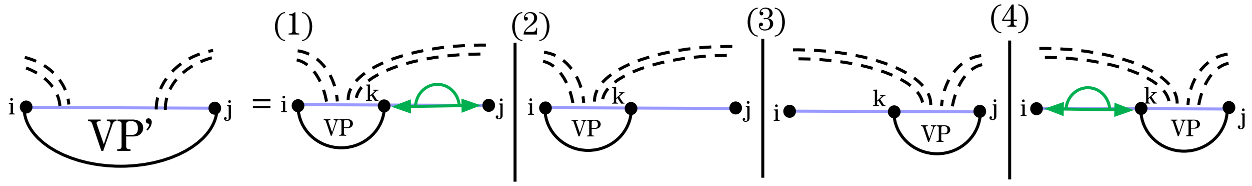

Figure 3: HFold's auxiliary VP' recurrence for parts of a multiloop that spans the band, where the next base pair of the band involves i or j. Cases 1 and 2 consume recursive substructures on the right of the base pair that crosses G. Cases 3 and 4 similarly consume recursive substructures on the left of the base pair that crosses G. Recall that the green bidirectional arrows with an arc represent a non-empty nested substructure handled by WI'.

4 Candidate Definitions

We define candidates for V, WMB and VP and the different structural loop contexts: external loop (W), multiloop (WM), pseudoloop (WI), or multiloops that span a band (WI'). Note that not all types of subproblems occur in all contexts in the recurrences of Spark. In the recurrences, we do not explicitly mention the type of candidates wherever it is clear from the recurrence.

Recall that $W_{i,j}$, $WM_{i,j}$, $WI_{i,j}$ and $WI'_{i,j}$ are defined only for weakly closed regions. In the definitions, we implicitly restrict conditions to the defined cases.

Definition 3 (V-candidate)**.** *The pair i, j is a V-candidate in loop context W (analogously WM, WI, WI′), if $V_{i,j} < W_{i,k-1} + W_{k,j}$ for all $i < k \leq j$.*

Note that the candidate criterion can be interpreted as $V_{i,j}$ is not optimally decomposable into two W subproblems of non-zero length. Candidates for pseudoloops are defined in a perfectly analogous way.

Definition 4 (WMB-candidate)**.** *The pair i, j is a WMB-candidate in loop context W (analogously WM, WI, WI′), if $WMB_{i,j} < W_{i,k-1} + W_{k,j}$ for all $i < k \leq j$.*

The definition of VP-candidates requires some adaption, since VP are defined for non-weakly closed regions. Note that $VP_{i,j}$ is defined only for regions that cross with G and the candidate criterion is limited to the defined cases.

Definition 5 (VP-candidate)**.** *The pair i, j is a VP-candidate in loop context WI (analogously WI'), if $VP_{i,j} < WI_{i,k-1} + VP_{k,j}$ for all $i < k \leq j$.*

Finally, BE candidates follow a different logic than the previous candidates. The number of valid $BE(i, i', j', j)$ is already strongly restricted, since band borders must bev base pairs in G. We restrict the candidates further based on perfect stacking.

Definition 6 (BE-candidates)**.** *The tuple (i, i', j', j) is a BE candidate iff $(i, j) \in G$, $(i', j') \in G$, $i < i' < j' < j$, $(i-1, j+1) \notin G$ and $(i'+1, j'-1) \notin G$.*

5 Spark Recurrences

5.1 $W_{i,j}$

$W_{i,j}$ is the MFE of all valid structures $R_{i,j}$ over region $[i, j]$. It is (finitely) defined, only if i and j are not covered by G and otherwise $W_{i,j} = \infty$. In the base case of an empty or singleton region, $i \geq j$, $W_{i,j} = 0$. For $i > j$, $W_{i,j}$ is given by the recurrence below:

$$W(i,j) = \min \begin{cases} (1) & V(i,j) \\ (2) & WMB(i,j) + P_s \\ (3) & W(i,j-1) \\ (4) & \min_{(k,j) \text{ is candidate}} W(i,k-1) + V(k,j) \\ (5) & \min_{(k,j) \text{ is candidate}} W(i,k-1) + WMB(k,j) + P_s \end{cases} \tag{1}$$

In Case 1, $R_{i,j}$ pairs i with j. In Case 2, i and j limit a pseudoknotted closed region in $[i, j]$. Since for defined W, this pseudoloop is exterior, the penalty P_s is added. In Cases 3–5, the structure $R_{i,j}$ can be decomposed into two independent, non-empty parts with either an unpaired base, loop-closed structure or pseudoloop-closed structure at the right.

5.2 $WI_{i,j}$

$WI_{i,j}$ is MFE of all valid structures $R_{i,j}$ given that $[i, j]$ is weakly closed, where energy is evaluated in the context of a pseudoloop. For non-weakly closed regions, detectable by cover$(i) \neq$ cover(j), $WI_{i,j} = \infty$. For empty regions, $i > j$, set $WI_{i,j} = 0$. Otherwise,

$$WI(i,j) = \min \begin{cases} (1) & V(i,j) + P_{ps} \qquad \text{if } (i,j) \in G \text{ or } \mathrm{bp}_G(i) = \mathrm{bp}_G(j) = -1 \\ (2) & WMB(i,j) + P_{sm} + P_{ps} \\ (3) & \min_{(k,j) \text{ is candidate}} WI(i,k-1) + V(k,j) + P_{ps} \\ (4) & \min_{(k,j) \text{ is candidate}} WI(i,k-1) + WMB(k,j) + P_{sm} + P_{ps} \\ (5) & WI(i,j-1) + P_{up} \end{cases} \tag{2}$$

In Case 1, i is paired with j in G or that both are unpaired and are instead paired in G'. In Case 2, i and j are ends of a pseudoknot in $R_{i,j}$. In Cases 3 and 4, there is a bifurcation in $R_{i,j}$ which splits into a region $[i, k-1]$ in WI and a base pair or pseudoknot (k, j). As the psuedoknot and base pair are within a pseudoloop, they are given the penalty P_{ps}. As Cases 2 and 4 are forming a new pseudoknot, they are given the penalty P_{sm} as well. Case 5 looks at an unpaired base on the right side.

5.3 $WI'_{i,j}$

$WI'_{i,j}$ is MFE of all valid non-empty structures $R_{i,j}$ of $[i, j]$ given that $[i, j]$ is weakly closed, where energy is evaluated in the context of a multiloop that spans a band. We set WI' to infinity, $WI'_{i,j} = \infty$, if cover$(i) \neq$ cover(j) or $i > j$.

$$WI'(i,j) = \min \begin{cases} (1) & V(i,j) + b' \\ (2) & WMB(i,j) + P_{sm} + b' \\ (3) & \min_{(k,j) \text{ is candidate}} c' * (k-i) + V(k,j) + b' \qquad \text{if empty(i,k)} \\ (4) & \min_{(k,j) \text{ is candidate}} c' * (k-i) + WMB(k,j) + P_{sm} + b' \qquad \text{if empty(i,k)} \\ (5) & WI'(i,j-1) + c' \qquad \texttt{if } \mathrm{bp}_G(j) = 0 \\ (6) & \min_{(k,j) \text{ is candidate}} WI'(i,k-1) + V(k,j) + b' \\ (7) & \min_{(k,j) \text{ is candidate}} WI'(i,k-1) + WMB(k,j) + P_{sm} + b' \end{cases} \tag{3}$$

In Case 1, i and j are either paired to each other by G or both unpaired in G, but paired in G'. In Case 2, i and j form a pseudoknot in $R_{i,j}$. In Cases 3 and 4, there is a bifurcation in $R_{i,j}$ which splits into a region $[i, k-1]$ made up of unpaired bases and a base pair or pseudoknot (k, j). In Case 5, there is a bifurcation in $R_{i,j}$ which splits into a region $[i, j-1]$ in WIP and an unpaired base. In Cases 6 and 7, there is a bifurcation in $R_{i,j}$ which splits into

a region $[i,k-1]$ in WIP and a base pair or pseudoknot (k,j). The cases are similar to that in $WI_{i,j}$ except the penalty for a base pair/pseudoknot is b' instead of P_{ps}. Unpaired bases within the region are also given the value c' instead of P_{up}.

5.4 $VP_{i,j}$

$VP_{i,j}$ is MFE of all valid structures $R_{i,j}$ of $[i,j]$. $VP_{i,j}$ is finitely defined only if some base of G crosses (i,j). It is infinite, i.e. $VP_{i,j}=\infty$, if (i,j) does not cross a base pair in G; if $i>j$; or if $\text{bp}_G(i)>0$ or $\text{bp}_G(j)>0$.

$$VP(i,j)=\min\begin{cases}(1) & WI(i+1,B'(i,j)-1)+WI(B(i,j)+1,j-1) \quad \text{if is\_covered}(i) \text{ and not is\_covered}(j)\\ (2) & WI(i+1,b(i,j)-1)+WI(b'(i,j)+1,j-1) \quad \text{if not is\_covered}(i) \text{ and is\_covered}(j)\\ (3) & WI(i+1,B'(i,j)-1)+WI(B(i,j)+1,b(i,j)-1)\\ & \quad +W(b'(i,j)+1,j-1) \quad \text{if is\_covered}(i) \text{ and is\_covered}(j)\\ (4) & e_{stp}(i,j)+VP(i+1,j-1) \quad \text{if } \text{bp}_G(i+1)=\text{bp}_G(j-1)=0\\ (5) & \min_{i<k<l<j} e_{intp}(i,k,l,j)+VP(k,l) \quad \text{if cover}(i)\text{=cover}(k) \text{ and cover}(l)\text{=cover}(j)\\ & \quad \text{and empty}(i,k) \text{ and empty}(l,j)\\ (6) & WV(i+1,j-1)+a'+2b' \quad i<k<\min(B'(i,j),b(i,j))\end{cases} \tag{4}$$

In Cases 1, 2, and 3, (i,j) is the only base pair which crosses the band borders in $[i,j]$. In Case 4, there is stacking base pair between a base pair at (i,j) and a base pair at $(i+1,j-1)$. In Case 5, there is an internal loop forming between a base pair (i,j) with another base pair at (k,l) of $R_{i,j}$. In Case 6, a similar to Case 5, there is base pair at (k,l) in $R_{i,j}$ that is within the base pair (i,j), but it allows closed regions in the regions $[i,k-1]$ and $[l+1,j]$. In this case, (i,j) closes a multiloop that spans a band.

5.5 $WV_{i,j}$

$WV_{i,j}$ is MFE of all valid structures $R_{i,j}$ over region $[i,j]$ given that $[i,j]$ is not weakly closed and contains at least two inner base pairs where one is a VP. WV represents the fragments of a multiloop that spans a band. The base cases are as follows:
$WV_{i,j}=\infty$ if (i,j) does not cross a base pair in G or $i>j$.

$$WV(i,j)=\min\begin{cases}(1) & \min\limits_{(k,j)\text{ is candidate}} WV^e(i,k-1)+V(k,j)+b' \quad \max(B(i,j),b'(i,j))+1<k<j\\ (2) & \min\limits_{(k,j)\text{ is candidate}} WV^e(i,k-1)+WMB(k,j)+P_{sm}+b' \quad \max(B(i,j),b'(i,j))+1<k<j\\ (3) & \min\limits_{(k,j)\text{ is candidate}} WI'(i,k-1)+VP(k,j)\\ (4) & \min\limits_{(k,j)\text{ is candidate}} WV(i,k-1)+V(k,j)+b' \quad \max(B(i,j),b'(i,j))+1<k<j\\ (5) & \min\limits_{(k,j)\text{ is candidate}} WV(i,k-1)+WMB(k,j)+P_{sm}+b' \quad \max(B(i,j),b'(i,j))+1<k<j\\ (6) & WV(i,j-1)+c'\end{cases} \tag{5}$$

In Cases 1 and 2, there is an empty region on the left side of the VP. To constrain the cases to one where a multiloop forms, candidate V or WMB is required to be on the right side. In Case 3, there is a closed region on the left side of the VP. This case does not constrict the right side, so it allows for unpaired bases or another closed region. In Cases 4 and 5, there is more than one base pair on the right side. In these cases, we are segmenting the region on the right to allow for multiple base pairs. As the energy relies on a $WV_{i,k-1}$, case 1, 2, or 3 must occur as well. In Case 6, there is an unpaired nucleotide on the right side.

5.6 $WV^e_{i,j}$

$WV^e_{i,j}$ is MFE of all valid structures $R_{i,j}$ over region $[i,j]$ given that $[i,j]$ is not weakly closed. WV represents a fragment of a multiloop that spans a band where the region on the left side is unpaired. $WV^e_{i,j}=\infty$ if (i,j) does not

cross a base pair in G or $i > j$.

$$WV^e(i,j) = \min \begin{cases} (1) \quad \min\limits_{(k,j) \text{ is candidate}} c' * (k-i+1) + VP(k,j) \quad \text{if empty}(i,k-1) \text{ and } i < k < \min(B'(i,j), b(i,j)) \\ (2) \quad WV^e(i,j-1) + c' \end{cases} \tag{6}$$

In Case 1, there is a VP forming at (k,j) with some number of unpaired nucleotides to the left of it.
In Case 2, there is an unpaired nucleotide on the right side. This allows there to be unpaired nucleotides to the right of the VP and before the V and WMB in Case 1 and 2 of $WV_{i,j}$.

5.7 $WM_{i,j}$

$WM_{i,j}$ is MFE of all valid structures $R_{i,j}$ over region $[i,j]$ given that $[i,j]$ is weakly closed, is not empty, and i and j are within a multiloop. $WM_{i,j} = \infty$ if $i > j$; otherwise

$$WM(i,j) = \min \begin{cases} (1) \quad WM^p(i,j) \\ (2) \quad V(i,j) + b \\ (3) \quad WMB(i,j) + b + P_{sm} \end{cases} \tag{7}$$

Case 1 occurs when the region can be decomposed into two independent subparts. Case 2 occurs if there is a base pair at (i,j). Case 3 occurs if there is a pseudoknot at (i,j).

5.8 $WM^p_{i,j}$

$WM^p_{i,j}$ is MFE of all valid structures $R_{i,j}$ over region $[i,j]$ given that $[i,j]$ is weakly closed, is not empty, and i and j are within a multiloop. $WM^p_{i,j} = \infty$ if $i > j$; otherwise

$$WM^p(i,j) = \min \begin{cases} (1) \quad WM^2(i,j) \\ (2) \quad \min_{i<k<j} \quad c * (k-i) + V(k,j) + b \\ (3) \quad \min_{i<k<j} \quad c * (k-i) + WMB(k,j) + b + P_{sm} \\ (4) \quad WM(i,j-1) + c \end{cases} \tag{8}$$

Case 1 occurs when the region $[i,j]$ can be decomposed into two independent closed regions. Case 2 and 3 occurs if there is a base pair or pseudoknot occurring within the region $[i,j]$ at (k,j) and there are unpaired bases between i and k. Case 4 occurs if there is an unpaired base on the right side.

5.9 $WM^2_{i,j}$

$WM^2_{i,j}$ is MFE of all valid structures $R_{i,j}$ over region $[i,j]$ given that $[i,j]$ is weakly closed and enclose at least two base pairs. WM^2 represents the multiloop fragments within a multiloop. $WM^2_{i,j} = \infty$ if $i > j$; otherwise

$$WM^2(i,j) = \min \begin{cases} (1) \quad WM^2(i,j-1) + c \\ (2) \quad \min_{(k,j) \text{ is candidate}} \quad WM(i,k-1) + V(k,j) + b \\ (3) \quad \min_{(k,j) \text{ is candidate}} \quad WM(i,k-1) + WMB(k,j) + b + P_{sm} \\ (4) \quad \min_{(k,j) \text{ is candidate}} \quad c * (k-i) + WMB(k,j) + b + P_{sm} \end{cases} \tag{9}$$

Case 1 occurs if there is an unpaired base on the right side. Case 2 and 3 occur if the the region $[i,j]$ can be decomposed into two subparts where (k,j) forms a base pair or a pseudoknot and [i,k-1] forms a weakly closed region Case 4 occurs if there is a single pseudoknot which accounts for the 2 base pairs needed for a multiloop (allows pseudoknotted multiloops where there is one pseudoknot only).

5.10 $V_{i,j}$

$V_{i,j}$ is the MFE of all valid structures $R_{i,j}$ over region $[i,j]$ given that $[i,j]$ is weakly closed and (i,j) closes a base pair. $V_{i,j} = \infty$ if $i > j$, or $[i,j]$ is not weakly closed, or i does not pair with j.

$$V(i,j) = \min \begin{cases} (1) & \mathcal{H}(i,j) \\ (2) & \min_{\substack{i<k<l<j \\ k-i+j-l<M}} \mathcal{I}(i,k,l,j) + V(k,l) \\ (3) & WM^2(i+1,j-1) + a + b \end{cases} \tag{10}$$

Note that the size of interior loops is limited by M. Case 1 occurs if (i,j) closes a hairpin. Case 2 occurs if (i,j) closes and internal loop with a nested base pair at (k,l). Case 3 occurs if (i,j) closes a multiloop comprised of the region [i+1,j-1].

5.11 $WMB_{i,j}$

$WMB_{i,j}$ is the MFE of all valid structures $R_{i,j}$ over region $[i,j]$ given that $R_{i,j}$ is a density-2 pseudoloop. $WMB_{i,j} = \infty$ if $i > j$, otherwise

$$WMB(i,j) = \min \begin{cases} (1) & P_b + \min_{\substack{(\ell,\ell',j',j) \text{ is BE-candidate} \\ i<\ell}} BE(\ell,\ell',j',j) + WMB^{\mathrm{A}}(i,j'-1) \\ (2) & WMB'(i,j) \end{cases} \tag{11}$$

In Case 1, j is the right border of a band in G, which crosses a pseudoloop prefix. The band penalty for the band of G corresponding to BE is added. Structures where j is unpaired in G are delegated to WMB' by Case 2.

5.12 $WMB^{\mathbf{A}}_{i,j}$

$WMB^{A}_{i,j}$ is the MFE of all valid structures $R_{i,j}$ over region $[i,j]$ given that $R_{i,j}$ is a subpart of density-2 pseudoloop. $WMB^{A}_{i,j} = \infty$, if $i > j$; otherwise

$$WMB^A(i,j) = \min \begin{cases} (1) & \min_{(k,j) \text{ is candidate}} \quad WMB^A(i,k-1) + V(k,j) \\ (2) & \min_{(k,j) \text{ is candidate}} \quad WMB^A(i,k-1) + WMB(k,j) \\ (3) & WMB^A(i,j-1) + P_{up} \\ (4) & WMB'(i,j) \end{cases} \tag{12}$$

Case 1, 2, and 3 occurs if we have called Case 1 of WMBP or case 2 of WMB and we are trying to decompose the $WMB^{\mathrm{A}}_{i,j}$ term into a shorter WMBA and a base pair, pseudoknot, or unpaired base. Note that the candidates are only considered if $[k,j]$ forms a weakly closed region. In these cases, the decomposed WMBP term is still enclosed by the same band as $WMB^{\mathrm{A}}_{i,j}$. Case 4 occurs if we have decomposed the right side of the WMBA such that j is part of a psuedoknotted base pair in the region $[i,j]$. In this case we move to WMBP where we break off the VP term.

5.13 $WMB'_{i,j}$

$WMB'_{i,j}$ is the MFE of all valid structures $R_{i,j}$ over region $[i,j]$ given that $R_{i,j}$ is a subpart of density-2 pseudoloop and j forms a pseudoknot with some base k. The base cases are as follows:
$WMB'_{i,j} = \infty$ if $i > j$

$$WMB'(i,j) = \min \begin{cases} (1) & 2 * P_b + \min_{\substack{i<k<\min(j,b(i,j)) \\ \text{not } isCovered(G,k) \\ (k,j) \text{ is candidate}}} BE(\mathrm{bp}_G(B(k,j)), \mathrm{bp}_G(B'(k,j)), B'(k,j), B(k,j)) + \\ & \quad WMB^A(i,k-1) + VP(k,j) \\ (2) & P_b + VP(i,j) \\ (3) & 2 * P_b + \min_{\substack{i<k<\mathrm{bp}_G(i) \\ (k,j) \text{ is candidate}}} BE(i, b'(i,k), \mathrm{bp}_G(b'(i,k), \mathrm{bp}_G(i)) + \\ & \quad WI(b'(i,k)+1, k-1) + VP(k,j) \end{cases} \tag{13}$$

In Case 1, j forms a band in G' but there is still more of the structure to be decomposed. The VP handles the band in G' and is given a band penalty. The band surrounding the right most side of VP is handled by BE and is also given a band penalty. The rest of the structure is then further decomposed by a WMB' term.
In Cases 2 and 3, $WMB'_{i,j}$ is the final decomposition and this iteration of WMBP yields the result. Case 2 occurs if i pairs with j in G'. This is handled by VP and is given a band penalty for the band formed by VP. In case 3, there are two bands formed by the BE term and the VP term. The WI term handles the region between $bp_{i,j}$ and the beginning of the VP term.

5.14 $BE_{i,k,l,j}$

$BE_{i,k,l,j}$ is the MFE of the band closed by $(i,j) \in G$ and whose innermost base pair is $(k,l) \in G$ such that $i \leq k \leq l \leq j$. $BE_{i,k,l,j} = \infty$, if these conditions do not hold. For $i < j$, $BE_{i,i,j,j} = 0$. Otherwise,

$$BE(i,k,l,j) = \min \begin{cases} (1) & e_{stp}(i,j) + BE(i+1,k,l,j-1) \\ (2) & e_{intp}(i,m,n,j) + BE(m,k,l,n), \quad \text{if } (i,j) \text{ and } (m,n) \text{ close an the interior loop of } G \\ (3) & WI'(i+1,m-1) + BE(m,k,l,n) + WI'(n+1,j-1) + a' + 2b' \\ (4) & c' * (m-i-1) + BE(m,k,l,n) + WI'(n+1,j-1) + a' + 2b' \\ (5) & WI'(i+1,m-1) + BE(m,k,l,n) + c' * (j-n-1) + a' + 2b', \end{cases} \tag{14}$$

Note that in Cases 2–5, there is a unique $(m,n) \in G$, $i < m \leq k, l \leq n < j$, that yields a finite result. Using only linear space, this (m,n) is determined in constant time.

In Case 1, (i,j) is stacked with $(i+1,j-1) \in G$. In Case 2, (i,j) forms an internal loop with base pair $(m,n) \in G$. In Case 3, (i,j) pairs and there is some (m,n) which forms the next part of the band. This case allows closed regions in the space between $[i,m]$ and $[n,j]$. Case 4 handles a similar case to case 3 except that the region between $[i,m]$ is empty instead of closed. Case 5 handles a similar case to case 3 except that the region between $[l,j]$ is empty instead of closed.

Linear space for storing WI'. The required WI' for BE can be stored in linear space since they are defined by consecutive ends of outer and inner base pairs of multiloops of G. Note that this is the only other use of WI' apart from the WI' recurrence itself, which also gets along with linear space.

6 Correctness of Spark-specific sparsification

Lemma 4 (Correct sparsification of WMB)**.** *The original and sparse recurrences of WMB are equivalent.*

Proof. We begin by restating the WMB recurrence of HFold:

$$WMB_{\text{HFold}}(i,j) = \min \begin{cases} (1) & P_b + \min_{\text{bp}_G(j)<k<j} \quad BE(\text{bp}_G(j), \text{bp}_G(B'(k,j)), B'(k,j), j) + \\ & \qquad WMB'(i,k-1) + WI(k, B'(k,j)-1) \\ (2) & WMB'(i,j). \end{cases}$$

We have to show the equivalence of its Case 1 to the first case of Eq. 11

$$P_b + \min_{\substack{(\ell,\ell',j',j) \text{ is BE-candidate} \\ i<\ell}} BE(\ell,\ell',j',j) + WMB^{\text{A}}(i,j'-1).$$

Let k^* denote the optimal k in a finitely defined $WMB_{\text{HFold}}(i,j)$, Case 1. Let $\ell := \text{bp}_G(j)$, $j'^* := B'(k^*,j)$, and $\ell'^* := \text{bp}_G(j'^*)$. Then, we can show that (ℓ,ℓ'^*,j'^*,j) is a BE-candidate with $i < \ell$. By definition (Def. 6), under the given conditions, it is a BE-candidate if and only if the base pairs $(\ell-1,j+1)$ and (ℓ'^*+1,j'^*-1) do not exist in G: 1) $(\ell-1,j+1) \in G$ would contradict $[i,j]$ weakly closed, holding by definition of WMB, and $i \leq \ell-1$. 2) (ℓ'^*+1,j'^*-1) would contradict $[k^*,j'^*-1]$ weakly closed, holding by definition of WI, and $\ell'^*+1 < k^*$.

Therefore, (ℓ,ℓ'^*,j'^*,j^*) is considered as candidate in the minimization of Eq. 11, Case 1. By optimality of $WMB^{\text{A}}(i,j'^*-1)$, $BE(\ell,\ell'^*,j'^*,j) + WMB'(i,k^*-1) + WI(k^*,j'^*-1) \geq BE(\ell,\ell'^*,j'^*,j) + WMB^{\text{A}}(i,j'^*-1)$, which implies the claim due to optimality of the l.h.s.

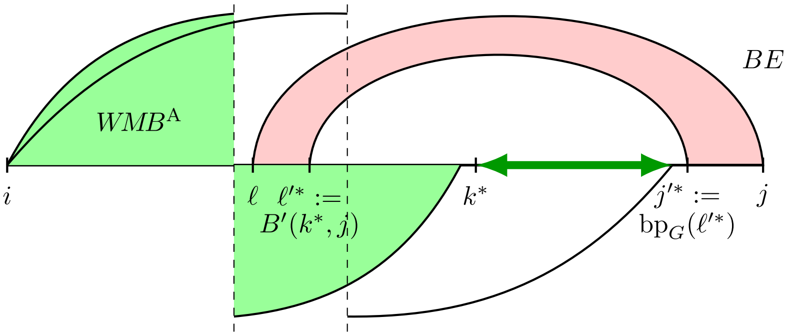

□

Lemma 5 (Correct sparsification using VP-candidates in WMB')**.** *The sparsified minimizations of recurrence WMB' are equivalent to the unsparsified ones. The required BE-values are limited to candidates or have left end i.*

Proof. One can show the correctness for Cases 1 and 3 independently. We show that it is sufficient to consider only VP-candidates *and* the BE-entries in Case 1 are candidates.

Case 1. Let k^* be the optimal k of Case 1; assume (k^*, j) is not a candidate. Then, there must be a $k > k^*$, such that $VP(k^*, j) > WI(k^*, k-1) + VP(k, j)$. Since then $[k^*, k-1]$ is weakly closed, $r := B(k^*, j) = B(k, j)$ and $r' := B'(k^*, j) = B'(k, j)$. Consequently,

$$\begin{aligned}
&BE(\text{bp}_G(B(k^*, j)), \text{bp}_G(B'(k^*, j)), B'(k^*, j), B(k^*, j)) + WMB^{\text{A}}(i, k^* - 1) + VP(k^*, j) \\
>&BE(\text{bp}_G(r), \text{bp}_G(r'), r', r) + WMB^{\text{A}}(i, k^* - 1) + WI(k^*, k - 1) + VP(k, j) \\
\geq&BE(\text{bp}_G(r), \text{bp}_G(r'), r', r) + WMB^{\text{A}}(i, k - 1) + VP(k, j),
\end{aligned}$$

contradicting the optimality of k^*.

Moreover, $(\text{bp}_G(r), \text{bp}_G(r'), r', r)$ is a BE-candidate, since

- $(\text{bp}_G(r) - 1, r + 1) \in G$ contradicts the maximality of $r = B(k^*, j) < j$ (see Def. of B). We can rule out $r = j$ since j is unpaired in G in valid $VP(i, j)$.
- $(\text{bp}_G(r') + 1, r' - 1) \in G$ contradicts the minimality of $r' = B'(k^*, j) > k^*$, We rule out $r' = k^*$, since k^* is unpaired for valid $VP(k^*, j)$.

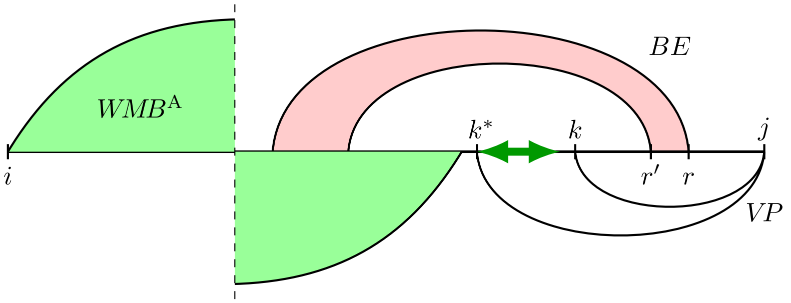

Case 3. The case is shown similarly. Let k^* be optimal and assume that $VP(k^*, j)$ is not a candidate since $VP(k^*, j) > WI(k^*, k-1) + VP(k, j)$. Then, since $[k^*, k-1]$ is weakly closed, $b(i, k^*) = b(i, k)$ and $b'(i, k^*) = b'(i, k)$, so that

$$\begin{aligned}
&BE(i, b'(i, k^*), \text{bp}_G(b'(i, k^*), \text{bp}_G(i)) + WI(b'(i, k^*) + 1, k - 1) + VP(k^*, j) \\
&> BE(i, b'(i, k), \text{bp}_G(b'(i, k), \text{bp}_G(i)) + WI(b'(i, k) + 1, k^* - 1) + WI(k^*, k - 1) + VP(k, j) \\
&\geq BE(i, b'(i, k), \text{bp}_G(b'(i, k), \text{bp}_G(i)) + WI(b'(i, k) + 1, k - 1) + VP(k, j)
\end{aligned}$$

contradicts the optimality of k^*. Here, the required BE value has left end i.

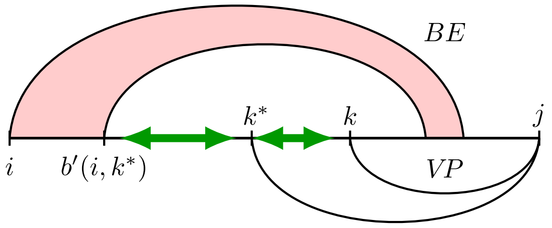

□

7 Benchmarking details

For benchmarking the hierachical folding algorithms and comparison to constraint folding, we generated realistically potentially relevant input structures by Algorithm 1 based on the Hotspots [Ren et al., 2005].

Algorithm 1 Input structure generation

for $k \leftarrow 1$ **to** n **do**
    for $l \leftarrow k$ **to** n **do**
        if k *pairs with* l **then**
            $e \leftarrow 0$
            $i \leftarrow k - 1$
            $j \leftarrow l + 1$
            while i *pairs with* j **do**
                $e \leftarrow e + e_{stack}(i, j)$
                $i \leftarrow i - 1$
                $j \leftarrow j + 1$
            end
        end
        $Hotspot \leftarrow (i, j, k, l, e)$
        $Hotspot\_list \leftarrow Hotspot$
    end
end
Sort Hotspot_list
return $Hotspot[0]$ ▷ Minumum energy stack structure

8 Additional Results

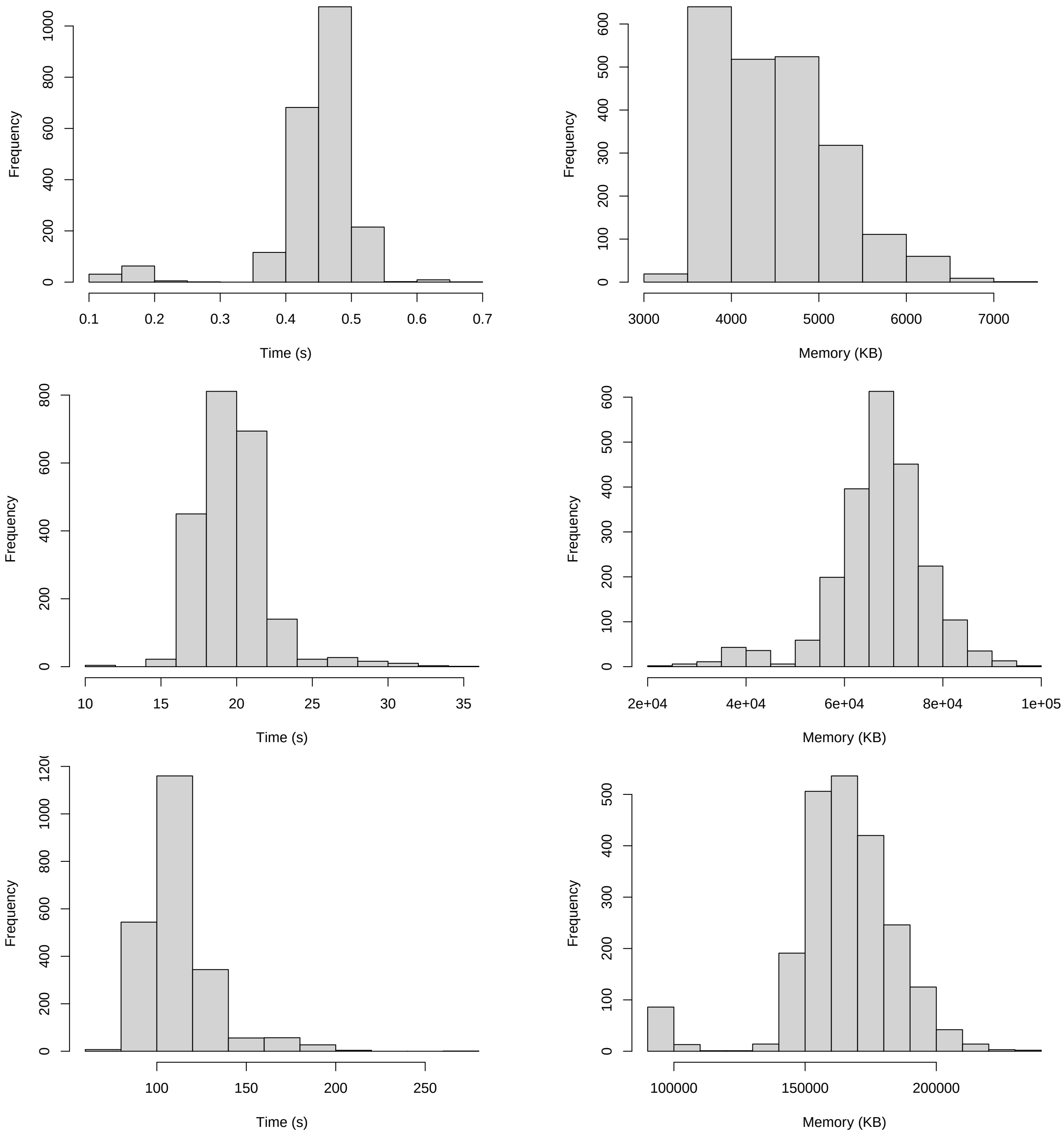

Figure 4: The effect of input constraints on time (left) and memory (right) for sequences of length 1000 (top), 5000 (middle), and 10000 (bottom)—based on 2200 sequence-structure combinations per sequence length.

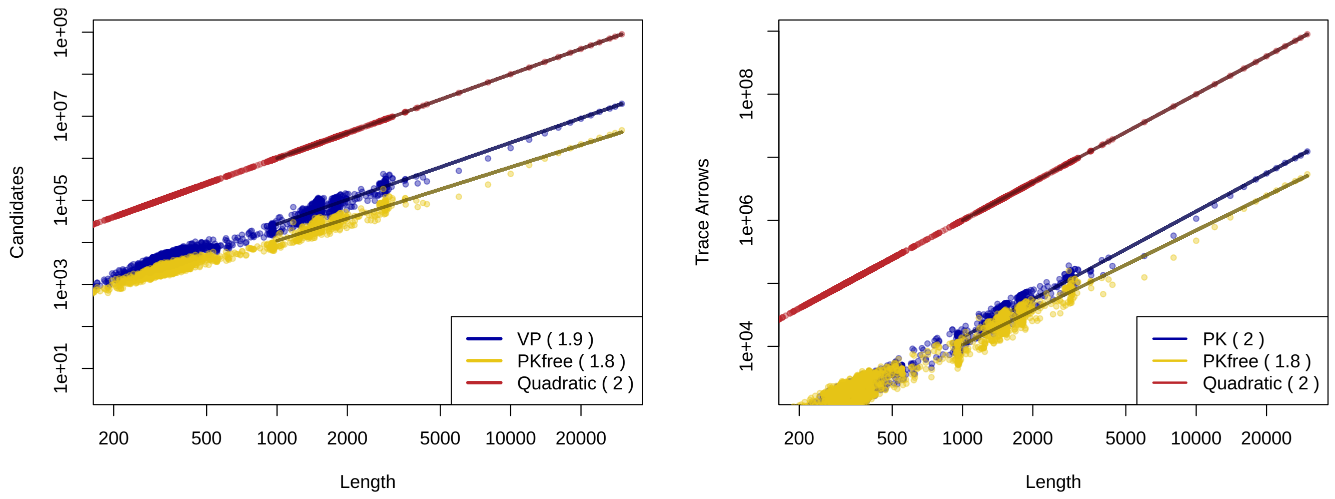

Figure 5: V candidates and trace arrows in Spark compared with pk-free folding by SparseRNAFolD. We analyze prefixes of the SARS-CoV-2 genome of varying lengths. Estimated scaling exponents are shown in parentheses; see main paper figures for details.

References

M. S. Andronescu, C. Pop, and A. E. Condon. Improved free energy parameters for RNA pseudoknotted secondary structure prediction. *RNA*, 16(1):26–42, 2010.

J. Fischer and V. Heun. A New Succinct Representation of RMQ-Information and Improvements in the Enhanced Suffix Array. In *Combinatorics, Algorithms, Probabilistic and Experimental Methodologies*, pages 459–470. Springer, Berlin, Germany, 2007. ISBN 978-3-540-74450-4. doi: 10.1007/978-3-540-74450-4_41.

H. Jabbari, A. Condon, and S. Zhao. Novel and efficient RNA secondary structure prediction using hierarchical folding. *J. Comput. Biol.*, 15(2):139–163, Mar 2008. doi: 10.1089/cmb.2007.0198.

B. Rastegari and A. Condon. Parsing nucleic acid pseudoknotted secondary structure: algorithm and applications. *J. Comput. Biol.*, 14(1):16–32, 2007.

J. Ren, B. Rastegari, A. Condon, and H. H. Hoos. Hotknots: heuristic prediction of RNA secondary structures including pseudoknots. *RNA*, 11(10):1494–1504, 2005.

C. Witwer, I. L. Hofacker, and P. F. Stadler. Prediction of consensus RNA secondary structures including pseudoknots. *IEEE/ACM Trans. Comput. Biol. Bioinf.*, 1(2):66–77, 2004.
